# Vasculature segmentation in 3D hierarchical phase-contrast tomography images of human kidneys

**DOI:** 10.1038/s41467-026-74050-8

**Published:** 2026-07-03

**Authors:** Yashvardhan Jain, Claire L. Walsh, Ekin Yagis, Shahab Aslani, Sonal Nandanwar, Yang Zhou, Juhyung Ha, Katherine S. Gustilo, Joseph Brunet, Shahrokh Rahmani, Paul Tafforeau, Alexandre Bellier, Griffin M. Weber, Peter D. Lee, Katy Börner

**Affiliations:** 1 Department of Intelligent Systems Engineering, Luddy School of Informatics, Computing, and Engineering, Indiana University, Bloomington, IN USA; 2 Department of Mechanical Engineering, University College London, London, UK; 3 Faculty of Medicine, Department of Surgery & Cancer, Imperial College London, London, UK; 4 UCL Centre for Medical Image Computing, London, UK; 5 European Synchrotron Radiation Facility, Grenoble, France; 6 Faculty of Medicine, National Heart and Lung Institute, Imperial College London, London, UK; 7 Department of Anatomy (LADAF), Université Grenoble Alpes, Grenoble, France; 8 Department of Biomedical Informatics, Harvard Medical School, Boston, MA USA

**Keywords:** Machine learning, Image processing, Scientific community, Software, Imaging

## Abstract

Efficient algorithms are needed to segment vasculature in new 3D medical imaging datasets at scale for research and clinical applications. Manual segmentation of vessels in images is time-consuming and expensive whereas computational approaches have limited accuracy. We organize a global machine learning competition, engaging 1,401 participants, to promote development of deep learning methods for 3D blood vessel segmentation in Hierarchical Phase-Contrast Tomography (HiP-CT) datasets. This paper presents a meta-analysis of the top-performing solutions, focusing on segmentation accuracy and morphological analysis. The competition and subsequent analysis reveal convergent methodological innovations: pseudo-labeling approaches that exploit data distributions, metrics and loss functions that optimize for vessel surface and topology, and multi-scale approaches that handle data heterogeneity. Additionally, the paper presents techniques for building deep learning models for the defined task, metrics to assess and compare algorithm performance, and a dataset with manually annotated and curated gold standard segmentations for future studies in blood vessel segmentation within HiP-CT imaging.

## Abstract (web summary)

The authors benchmark vascular segmentation in 3D HiP-CT images, revealing that no single metric captures quality. Top methods converge on pseudo-labeling and topology-aware losses, yet all struggle with microvascular connectivity breaks.

## Introduction

Because blood vasculature extends to all organs of the body, identifying vessels in medical images (vessel segmentation) is an important part of image processing in applications ranging from basic science research to clinical care.

As the vascular network is an inherently 3D and multi-scale spanning structure, imaging techniques that do not require physical subsampling to achieve high-resolution images across multiple scales are the ideal tool for vascular analysis^1^; Hierarchical Phase-Contrast Tomography (HiP-CT) is such a technique^2^. Leveraging the European Synchrotron Radiation Facility’s (ESRF) Extremely Brilliant Source (EBS), the world’s first high-energy fourth generation synchrotron source, HiP-CT enables imaging of intact human organs at unprecedented scale and resolution. HiP-CT can be used to map and quantify the arterial vascular network of an intact human kidney down to the arteriolar level^3^. Such large scale vascular network segmentations are of great utility for multiscale modeling of blood flow, where real/realistic micro-circulation models are coupled to lumped parameter or synthetic capillary level models to provide simulation of drug delivery and solute transport across tissues^4^. However, the creation of 3D models from HiP-CT data using gold standard manual segmentations is very time-consuming and labor-intensive. This labor-cost barrier impedes the broader utilization of 3D data to answer questions proposed by the biomedical community, including understanding exosome transport.

Having conducted initial studies into the challenges for application of machine learning (ML) models to HiP-CT vascular segmentation^5^, a global citizen science competition was organized on Google’s ML platform, Kaggle, to develop open-source machine learning solutions capable of automatically segmenting vascular networks of the human kidney in HiP-CT datasets. The Kaggle platform has been previously leveraged to collaboratively develop machine learning solutions for complex biomedical tasks, thereby advancing the field^6–9^. The competition presented in this paper focuses on 3D data, challenging teams to develop solutions to accurately segment blood vessels in HiP-CT images to create high-quality 3D models of vessels from organs such as the kidney. Such segmented 3D models can be used to characterize other morphological features of the networks such as length, diameter, branching angles, connectivity, tortuosity, inter-vessel distance, and can ultimately be used in multi-scale models of blood flow and solute delivery.

The two sponsors of this competition are the NIH Common Fund’s Human BioMolecular Atlas Program (HuBMAP) and Cellular Senescence Network (SenNet) Program. Both provide examples of how researchers can benefit from improved methods of vessel segmentation and serve as motivation for the competition:

1. HuBMAP is constructing a map of all the cells in the healthy adult human body. It envisions blood vasculature pathways serving as landmarks to describe the location of cells in organs and tissue^10–12^. In this Vasculature Common Coordinate Framework (VCCF)^13^, accurate vessel segmentation is essential to define these landmarks. As part of this work, HuBMAP has published an initial database of more than 900 blood vessels based on literature review and domain expert curation^14^. However, this needs to be linked to experimental data for validation and to extend it to microvasculature, where there are many gaps in existing knowledge.
2. SenNet studies senescent cells of the human body—in health and in disease—across the lifespan^15,16^. A key challenge is understanding the secretome, i.e., the set of proteins these cells secrete into the extracellular space and/or the bloodstream. Exosomes—a subcategory of secretomes—are membrane-bound extracellular vesicles used to transport diverse cargo to neighboring cells and to other organs. However, understanding the generation, transportation, and ingestion of exosomes requires data on the human blood vasculature—the main exosome transport system. By analyzing this complex highway of blood flow which includes determining the pathway, throughput/vessel size, and 3D orientation in space of the blood vasculature, we aim to contribute to the understanding of exosome diffusion via the bloodstream, how exosomes can play roles in organ-to-organ communication, and where exosomes might aggregate due to branching or tortuosity of the vessels—analogous to river sediments^17–19^.

The challenges of developing automated vascular segmentation for HiP-CT data are threefold–data scarcity, connectivity preservation, and dataset heterogeneity–and represent fundamental problems in applying deep learning to novel biomedical imaging modalities, extending well beyond the specific case of kidney vasculature. This paper presents how specialists across HiP-CT imaging, renal physiology, ML engineers, and data engineers collaboratively set up the curated HiP-CT 3D datasets, defined the competition metrics, and deployed the challenge on an established open competition platform to benchmark performance and systematically explore the solution space at scale. By leveraging a global community of participants resulting in many thousands of independent submissions, the competition provided a broad overview of methodological strategies, allowing the organizers to identify common architectural patterns, training paradigms, and preprocessing approaches shared across the majority of top-performing models, while also detecting specific, innovative, or fundamentally novel techniques that diverged from prevailing trends yet achieved competitive performance. Through analysis of the discussion forums and the top-5 winning solutions, the competition framework therefore provides both consensus-driven best practices and insight into creative methodological advances that can be utilized by the broader community interested in addressing either the computational or biological challenges posed.

To enable a holistic evaluation of ML model performance, three additional analyses are provided: quantitative (based on additional metrics), qualitative (based on visual analysis of predictions), and morphological (based on vasculature morphology features). Due to the novelty of HiP-CT data and lack of gold standard segmentations for vasculature in such data, the paper presents a curated dataset, relevant performance metrics, and performance scores for the top-5 solutions for future research in the domain, which can accelerate the augmentation of our knowledge of the blood vasculature system in support of answering key questions posed by the research community.

## Results

### Competition design

The “SenNet + HOA: Hacking the Human Vasculature in 3D” competition—running from November 7, 2023, through January 30, 2024—aimed to develop machine learning solutions for segmentation of blood vasculature in 3D HiP-CT scans of the human kidney. The imaging datasets were collected as part of the Human Organ Atlas effort (https://human-organ-atlas.esrf.eu). Vessel segmentation is a challenging task for various reasons: it is an imbalanced class problem (where prediction targets are disproportionately lower in volume compared to non-target background), there is a very low tolerance for error since small losses in segmentation connectivity can lead to large variations in simulated function^20^, there can be collapse or infilling of vessels during preparation which makes them challenging to segment^5^, there is wide image variability due to natural human anatomical variation and due to the nature of imaging post-mortem organs, and there exist relatively small training datasets due to the novelty of the HiP-CT imaging technique. Ahead of the competition, the authors conducted a study to investigate and document how these issues affected ML segmentation of vascular networks and also showcased the performance of a baseline nnU-Net^5,21^ model on HiP-CT data. The authors also examined a large range of metrics in order to analyze how different metrics may benefit certain types of segmentation models. This initial work highlighted the key areas that the competition participants should be focused on: (1) To develop solutions that could handle large 3D datasets and (2) To develop solutions that generalize to different HiP-CT datasets, i.e., that could account for variability in resolutions, image intensities, donors, and imaging conditions.

**Fig. 1:**
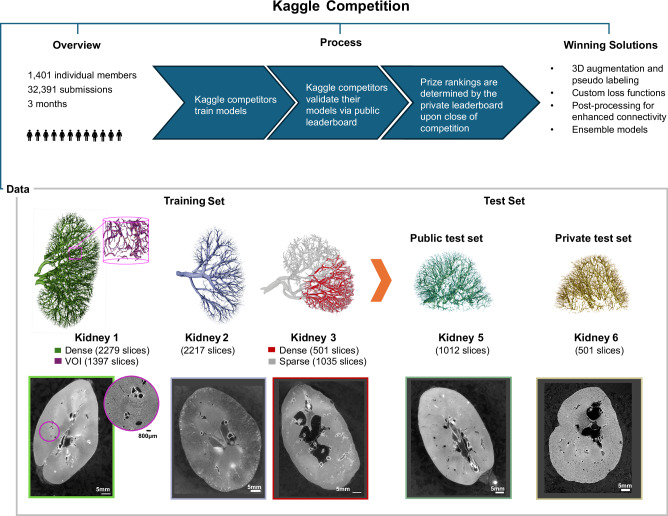
Competition overview. An overview of the competition setup and process, including 3D renderings of the gold standard labels and representative 2D slices for all data in the competition.

The HiP-CT datasets used in the competition were sourced from the kidneys of five adult donors. Table 1 provides an overview of the donor demographics, image and scanning metadata, and gold standard label metadata associated with each dataset (Full scanning and donor medical metadata can be found at https://human-organ-atlas.esrf.eu, DOI and link for each dataset are provided in Supplementary Table 1). Figure 1 gives an overview of the competition data and setup. The five 3D image volumes were split into training and test datasets. The training dataset contained four image volumes, including three whole kidney (~50 µm/voxel) datasets and one high resolution hierarchical Volume-of-Interest (VOI) dataset. Two of these whole kidney datasets contained a mix of sparse labels as well as dense labels (see *Methods*). The test set was further divided into a public test set and a private test set. The public test set contained one partial kidney volume with 50.28 µm/voxel data (1,013 slices). The private test set contained one partial kidney volume with 63.08 µm/voxel data (501 image slices), see *Methods* for details. It should be noted that the public test dataset was very similar to the training dataset, e.g., the same donor as kidney 1 (but left rather than right kidney) and collected on the same beamline BM05. By comparison, the private test data was from a different donor and collected with the same technique but on a different beamline, BM18. This choice of test data was made to reflect the challenges faced by the research teams, e.g., to create models from limited training datasets such that the models generalize effectively to new donors and are robust to changes in the imaging setup. In total, approximately 600 expert human hours were spent in segmentation and verification of the gold standard labels for the competition dataset, underscoring the time- and resource-consuming nature of the segmentation problem and the need for automated methods for the task.

**Table 1:** Competition dataset overview

Kaggle Donor ID	Donor ID	Segmentation labels	Voxel size (µm)	Image z-slices	Dataset usage	Beamline	Sex	Age
**1**	LADAF 2021-17-Right-Kidney	Whole kidney densely labeled	50	2279	Training	BM05	M	63
**1**	LADAF 2021-17-Right-Kidney	Whole VOI densely labeled	5.2	1397	Training	BM05	M	63
**2**	S-20-28-Kidney	Whole kidney sparsely labeled (65% of vessels)	50	2217	Training	BM05	M	84
**3**	LADAF-2020-27-Kidney-2	Whole kidney part densely, remainder sparsely, labeled, (85% of vessels)	50.16	501 dense 1035 sparse	Training	BM05	F	94
**5**	LADAF-2021-17-Left-Kidney	Partial whole kidney densely labeled	50.28	1012	Public test	BM05	M	63
**6**	LADAF-2022-13-Bottom-Kidney	Partial whole kidney densely labeled	63.08	501	Private test	BM18	M	85

Competition dataset details, including metadata and donor demographics.

The challenges associated with the creation of high quality gold standard labels for training ML models are well known^22^. However, with new modalities such as HiP-CT, this poses a particular challenge as there is no large pool of potential expert curators who are familiar with the data type and the particular artifacts that can be present. In order to acknowledge the challenge with creating this gold standard, we created a labeling protocol involving three independent curators (see *Methods* for details). Using this protocol, we were able to provide both dense labeling (i.e., where the third, quality control, curator found 100% of vessels were labeled), and to provide sparse labeling with an estimate of the sparsity. This allowed some teams, e.g., Team 3, to use the sparse labels effectively as training data within a pseudo-labeling context. This was critical in the competition design as it is relatively fast for an experienced curator to sparsely label an image volume but highly time consuming to label it densely.

In addition, a high-resolution VOI was provided, which is a subset of the lower resolution whole kidney training dataset that can be rigidly registered to the whole kidney dataset to provide more accurate labels for smaller vessels. Previously, we have shown how these higher resolution VOIs can be used to validate manual segmentation in the lower resolution dataset^3^. Interestingly, none of the teams attempted to utilize or extend this multi-resolution approach to improve the segmentation of small vessels, although teams that used this dataset as additional training data, such as Team 1, found a greater proportion of the smaller vessels.

In addition to the competition data, participants were allowed to use publicly available external datasets. Some teams used other HiP-CT datasets (gold standard labels absent) available via the Human Organ Atlas data portal (https://human-organ-atlas.esrf.eu) to help improve the performance of their solutions. All inference code was submitted via the Kaggle submission portal and was run on the public and private test sets within the Kaggle infrastructure, leading to team rankings on the public and private leaderboards (LB), respectively (see *Methods*). Throughout the competition, the private LB scores and rankings are not visible to the participants, which forces them to use the public LB scores and rankings as a validation dataset for their experiments. The test datasets are not visible to the participants, but they are provided with 3 *z*-slices each from both test sets as examples. They are also provided with the voxel resolution for both test sets.

Algorithm performance was judged based on the Normalized Surface Dice (NSD) metric as previously proposed by the Google DeepMind team^23,24^, with tolerance threshold set to 0 (see *Methods*). At the end of the competition, the top-5 teams on the final private leaderboard won the performance prizes and the associated prize money (see *Methods*).

**Fig. 2:**
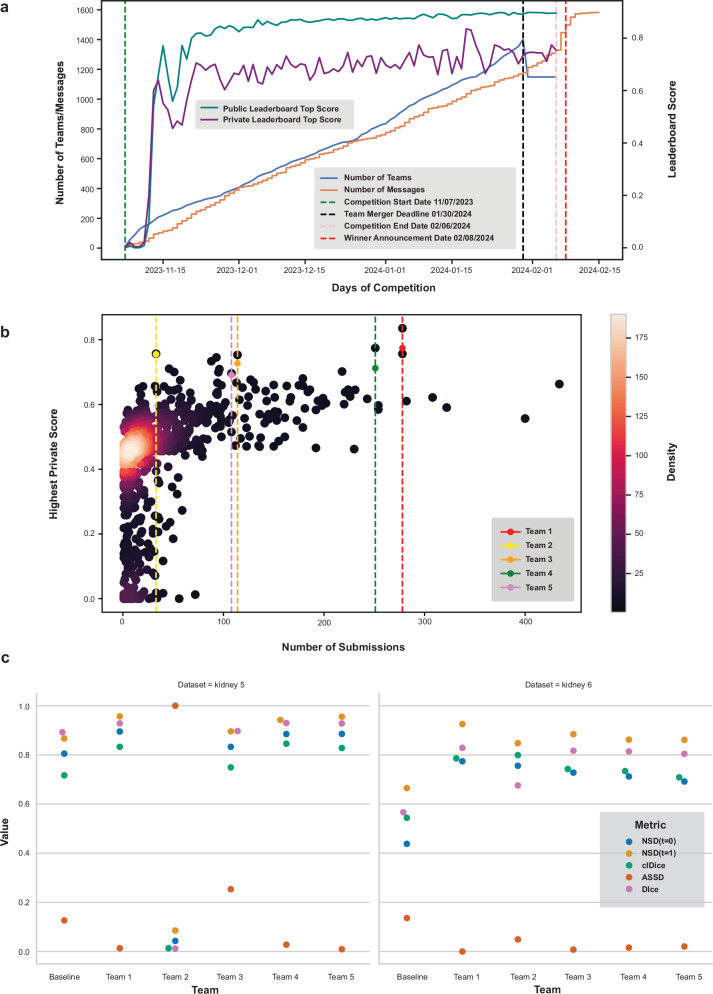
Competition dynamics and quantitative analysis. **a** Number of teams and messages, and leaderboard high scores per day over competition period. **b** Number of submissions vs. highest private leaderboard score for each of the 1149 teams as a heat scatter. **c** Scores for competition metric and additional metrics for top-5 teams on both test sets. ASSD is normalized between 0 and 1 for visualization (lower is better). Source data are provided as a Source Data file (see *Data Availability*).

The competition observed participation by 1401 individual members divided across 1149 teams from 78 countries. There were 204 members that participated for the first time in a Kaggle competition, five of which were in the top-100 on the final private leaderboard. There were 32,391 unique code submissions, 500 public notebooks created, 756 discussion comments, and 200 discussion forum topics. There was also a separate Discord server created for the competition where teams engaged in more informal discussions throughout the competition, although this was not monitored by the hosts/authors. Figure 2a graphs the changes in the leaderboard scores, forum activity, and number of participants during the 3-month period of the competition. Figure 2b shows the number of submissions versus the highest private score for all teams.

### Overview of winning solutions

The competition revealed three primary methodological innovations that collectively address the fundamental challenges of vascular segmentation in novel imaging modalities with limited annotated data: (1) sophisticated pseudo-labeling strategies that utilize both sparse competition labels and unlabeled external datasets, (2) loss functions that leverage and optimize vascular-specific properties such as surface boundaries, topology, and connectivity, and (3) multi-scale spatial processing strategies that leverage the feature heterogeneity of HiP-CT data. All teams, especially the top-5 winning teams, employed some variation and combination of these methodologies to optimize their solutions, underscoring their importance for the vascular segmentation task.

#### Pseudo-labeling as the key optimization strategy

With the limited expert-annotated gold standard training data, all top-5 winning teams except Team 2 employed some variation of pseudo-labeling strategies to expand their training datasets. However, their approaches differed in implementation: Team 4 applied basic pseudo-labeling to external datasets using models trained on competition data; Team 5 developed a progressive four-step refinement approach with soft pseudo-labels (i.e., probability outputs instead of binary masks) to reduce error propagation from false positives; Team 3 implemented a more advanced strategy with an iterative sparse-to-dense refinement process that used domain knowledge about distributional similarity between kidneys from the same donor. Critically, Team 3’s approach incorporated pseudo-label quality control by using dynamic thresholding to match the known sparsity levels of partially-labeled datasets. This progression from basic to domain-aware pseudo-labeling strategies demonstrates how citizen science competitions can enable rapid exploration of the solution space of a challenge to propose different methodologies.

#### Handling feature heterogeneity of HiP-CT imaging

Since different HiP-CT datasets have substantial resolution and intensity differences, teams had to use multi-scale spatial processing strategies to optimize their solutions. Team 3 applied heavy intensity augmentation and emulated test set magnification to match resolution differences. Team 4 implemented percentile-based stack-wise normalization to standardize intensity ranges while preserving relative contrast. Team 5 performed 3D trilinear interpolation post-inference to account for resolution differences between training and test datasets. Data heterogeneity and pseudo-labeling strategies highlight that model performance gains are increased when the unlabeled and gold standard datasets share the same distributional characteristics.

#### Model architecture and dimensionality strategies

The competition highlighted fundamental trade-offs in 3D medical image processing, with all top-5 teams employing U-Net^25^ variants customized for the vascular segmentation task. Team 2 used a 3D U-Net^26^ approach that would provide optimal spatial context but had to be constrained to small input patches (128 × 128 × 32 voxels) due to GPU memory limitations, which then required extensive training (700 epochs over 2 weeks) and limited the model’s ability to capture larger-scale vascular patterns. In contrast, Team 1 used a 2.5D approach–stacking three consecutive slices as input channels–which enabled processing of much larger patches (1536 × 1536 pixels) while retaining limited 3D context. Additionally, Team 1 incorporated an extra stem block specifically designed to preserve high-resolution features for small vessel detection and used ConvNeXt-tiny^27^ as the encoder backbone. Team 3 and Team 5 adopted multi-axis 2D strategies, training separate models along orthogonal planes (*xy*, *yz*, *xz*), enabling 512 × 512 patch sizes while implicitly capturing 3D information through ensemble aggregation. Notably, Team 3 employed MaxViT-Large-512^28^ as the encoder backbone, leveraging the dual capabilities of Convolutional Neural Networks^29^ (CNNs) and Transformers^30^; while CNNs can efficiently extract local features, Transformers can capture global contextual information, thereby enabling the model to combine both local and global features to accurately segment complex vascular structures^31^. Team 4 used a hybrid approach and combined 2D and 3D models along with a weighted sampling approach. These diverse strategies highlight that the optimal model architectures depend on computational constraints, desired spatial context, and vascular scale priorities (i.e., small peripheral vessels vs. large central vessels). Furthermore, larger patch sizes reduce boundary artifacts introduced during sliding-window inference.

#### Strategies for loss function design

A key insight from the competition was that teams gained substantial performance improvements by designing loss functions that optimized directly for the evaluation metric (NSD at *t* = 0) and topology preservation. Team 1 developed a computationally efficient custom Marching Cube Loss (MCL) that directly optimizes surface-level accuracy, and combined it with focal loss^32^, dice loss^33^, and boundary loss^34^, increasing their score from 0.534 to 0.682 on the private LB. Team 4 employed BoundaryDOULoss^35^ for both 2D and 3D models (with a 3D-specific variant), achieving a 5% improvement on the private LB from this loss function choice. Team 5 implemented a boundary-weighted binary cross-entropy loss that doubled the loss contribution from boundary pixels (using weight = 0.9) to sharpen boundary predictions, and combined it with dice loss and focal loss. In contrast, Team 2 optimized their model using focal loss but achieved topology preservation through post-processing. These strategies highlight that when designing loss functions for vascular segmentation, it is important to consider surface and topology optimization collectively to reduce connectivity errors.

#### Synthetic data generation via data augmentation techniques

To overcome the issue of limited training data, in addition to pseudo-labeling, data augmentation techniques were essential for model optimization and generalization. Team 1 implemented an efficient 3D rotation augmentation strategy that enabled on-the-fly geometric transformations. Team 2 generated over 7000 patches per epoch using 3D affine transformations (rotation, scaling, and shearing), effectively expanding the training set by orders of magnitude. Team 4 adapted the CutMix^36^ technique for vascular segmentation with a critical domain-specific constraint, i.e., only slices from the same anatomical view could be mixed, limiting anatomically impossible vascular configurations. Additionally, Team 4 implemented a weighted sampling scheme based on label density, ensuring the model saw proportionally more examples from densely-labeled datasets during training. Team 3 implemented heavy intensity augmentation strategies to enhance model robustness by tackling intensity variations across different datasets. Team 5 also implemented intensity contrast changes, flipping, and rotation-based augmentation techniques. Overall, these strategies highlight that efficient data augmentation techniques should be informed by computational and anatomical constraints of the target domain.

#### Advanced inference and ensemble strategies

In addition to the training strategies, inference strategies were similarly important for enhanced model performance. Team 1 processed data along three orthogonal axes with large sliding windows (3072 × 3072 pixels) and test-time augmentation (TTA), then ensembled predictions from models trained with and without 3D rotation. This strategy is particularly effective at mitigating the impact ring artefacts that are a feature of all computed tomography modalities. Team 4 implemented an ensemble strategy that combined 2D and 3D models with equal weighting, and a post-processing strategy to multiply 3D model predictions with 2D model-derived region-of-interest masks. Team 5 performed ensemble aggregation at the logit level (before sigmoid activation) rather than probability level, preserving more information for the final thresholding. Additionally, Team 5 applied a rescaling operation through three axes by trilinear interpolation directly on the 3D image, highlighting the importance of maintaining the voxel sizes of training and test datasets to be at a similar resolution. Team 2 implemented a connected component analysis using depth-first search to effectively remove false positive predictions in background regions and contributed to the model’s improved performance. Four out of the top-5 winning teams utilized ensemble models, demonstrating the strength of combining different model architectures for more robust predictions by averaging out individual model biases and effectively handling anomalies and noise in the data.

The innovative combination of architectural decisions, sophisticated loss functions and data augmentation, and inference-time strategies enabled the top-5 teams to develop high-scoring solutions in the competition. The key technical innovations–including Team 1’s custom Marching Cube Loss and extra stem block, Team 2’s topology-preserving post-processing, Team 3’s iterative pseudo-labeling, Team 4’s hybrid 2D and 3D model ensemble, and Team 5’s logit-level ensembling–are detailed further in *Methods*.

### Quantitative analysis of solutions

The top-winning team, Team 1, reached a NSD score of 0.774 on the final private LB and ranked first on the public LB with a score of 0.898, followed by 0.755 for Team 2, 0.727 for Team 3, 0.712 for Team 4, and 0.691 for Team 5. The top-5 teams made a total of 784 out of 32,391 submissions (2.42% of total submissions). Supplementary Table 2 lists public and private LB scores for all five winning teams, including the baseline nnU-Net model’s performance, and a brief summary of their solutions.

In comparing team rankings across public and private LBs, Team 1’s solution proved to be the most stable as it ranked at the top on both LBs. Team 4 and Team 5 were fairly stable: Team 4 rising by one place and Team 5 rising by five places on the private LB compared to the public LB. The solution by Team 2 was the most unstable, jumping 1022 places on the private LB, while Team 3 jumped by 568 places. Team 2’s solution was highly overfit to the private test set, and scored 0.04 NSD score on the public LB.

Additionally, the authors previously identified certain key metrics for the vessel segmentation problem after reviewing the existing literature^5^. These additional metrics—Dice Similarity Coefficient (Dice), Centerline Dice (clDice), NSD (tolerance = 1), and Average Symmetric Surface Distance (ASSD)—are computed for the predictions of the top-5 winning teams (see *Methods* for details on the metrics). All computed metrics for both test sets are provided in the Supplementary Table 3 and visualized in Fig. 2c.

The correlation between the models’ methodological characteristics and multi-metric generalization performance (Fig. 2c, Supplementary Table 3) can reveal the impact of different technical approaches to model robustness and segmentation quality. Team 1’s combination of 3D rotational augmentation, custom Marching Cube Loss (combined with other loss functions), and extra stem block achieved the most stable performance across both datasets. Team 2’s catastrophic failure on the public test set but dramatic improvement on the private test set suggests extended small patch 3D training might have led the model to overfit to a specific data distribution. Additionally, the depth-first search-based post-processing improved overall network connectivity as suggested by the high clDice score on the private test set. Team 3 shows degradation in performance on the private test set (compared to public test set) on NSD (*t* = 0) and Dice, but remains fairly stable for NSD (*t* = 1) and clDice, and actually shows a major improvement on ASSD; it suggests that while the model can predict most of the vessels (within some boundary tolerance), it has major boundary failures in isolated regions for the public test set which has less effect on the NSD but a major impact on the ASSD. This might be a result of the training strategy employed by Team 3 to primarily optimize for test set characteristics (such as resolution) via test set magnification and pseudo-labeling. Since data interpolation is generally approximate, it might explain the model’s decreased NSD performance at *t* = 0 but fairly stable performance at *t* = 1 (and clDice). Team 4 and Team 5 showed consistent results across all metrics except ASSD; both teams had similar scores, performing better on the public test set than private test set. Team 4 showed relatively stable ASSD across datasets, while Team 5’s ASSD increased moderately on the private test set. This suggests that while these two models don’t score the highest among the winning models, the strategies employed by these two teams resulted in relatively stable predictions that could potentially be improved by further training on more data. These multi-metric results demonstrate that robust generalization may be negatively impacted when models overfit dataset-specific characteristics through extensive training on limited data distributions, while successful approaches either employ loss functions targeting tubular vascular properties or use multi-metric optimization with balanced architectural strategies. Multi-metric evaluation that optimizes for vessel volume, surface, and connectivity is essential for better modeling.

A fundamental challenge in organizing such competitions that need to be scored based on a single metric is that changing the metric can change the outcome of leaderboards as well as the ability of the created solutions^37,38^. Considering this, it is valuable to see the correlation between public and private LB rankings as well as the effect of rankings based on different metrics. Subsequently, Kendall’s Tau^39^ (KT, see statistical analysis in *Methods*) is computed and a value of 0.0253 (*p* = 0.795) is found for rankings of private and public LB (top-50 teams). This highlights a high shake-up in rankings on both LBs, showing that, in general, solutions by top-50 participants had low generalizability. To check rank stability based on different metrics, KT is computed for all additional metrics with respect to the original competition metric. The KT for top-50 teams on the private test set based on NSD (tolerance=0) and clDice is 0.502 (*p* = 2.6839e-07), KT for NSD at tolerance 0 vs. 1 is 0.472 (*p* = 1.2773e-06), KT for NSD (tolerance = 0) and Dice is 0.387 (*p* = 7.0881e-05), and KT for NSD (tolerance = 0) and ASSD is 0.3877 (*p* = 7.0881e-05). Supplementary Table 3 shows all metric scores for top-50 teams for both test sets.

In addition to the competition data, the five winning models were also tested on an additional high-resolution VOI insert from donor 1 (6.5 µm/voxel, binned to 13 µm/voxel) post-competition. The relative performance of the models was similar to their performance on competition data, with Team 1 having the best performance in terms of NSD (tolerance = 0). Performance metrics scores are provided in Supplementary Table 4. The imaging configuration for this dataset is distinct from those of the overview (~50 µm/voxel) datasets and the very high resolution (5.2 µm/voxel) datasets, in terms of energy, filters, detector camera, magnification, propagation distance, etc., and thus the contrast and image intensity statistics are distinct from the other cases (i.e., this cannot be simplistically considered as a binned version of 5.2 µm/voxel dataset or an upsampled version of the 50 µm/voxel dataset). Additionally, this is a much larger dataset (1922 × 1922 × 5955 dimensions) and as such the Team 1 solution was able to handle the inference on this dataset with ease, but the Team 3 solution and Team 5 solution had to be modified to handle larger images to overcome Out Of Memory (OOM) issues. While these models can handle datasets with higher resolutions, especially Team 1 and Team 3 solutions, they would benefit further with more training data at higher resolution.

### Qualitative analysis of winning solutions

**Fig. 3:**
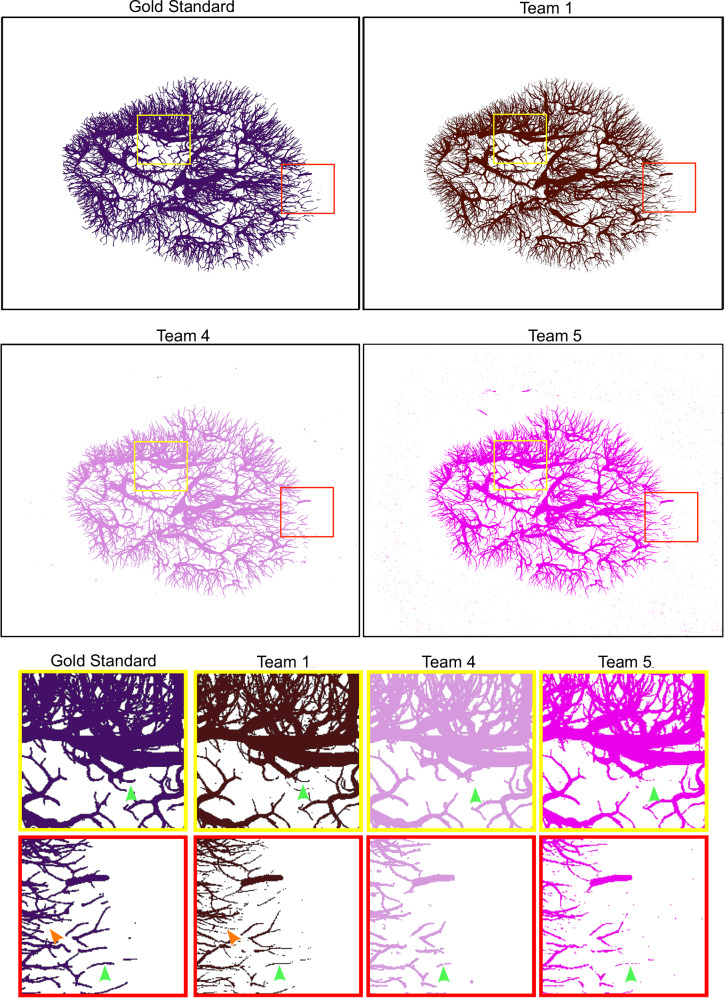
Maximum intensity projections for qualitative analysis. Maximum Intensity Projections (MIP) for the labels (private test set) for gold standard, Team 1 predictions, Team 4 predictions, and Team 5 predictions, with two insets per team in the yellow and red squares, respectively. Green arrows show the same location in each prediction highlighting the missing and unconnected vessels in Teams 1, 4 and 5. The orange arrows in the gold standard and Team 1 show an instance of a vessel predicted by Team 1’s model that is not in the gold standard but is, in fact, in the raw data.

Due to the low generalizability of solutions by Team 2 and Team 3, the qualitative and morphological analysis focuses on Teams 1, 4, and 5. Figure 3 shows a Maximum Intensity Projection (MIP) of the gold standard labels for the private test set alongside the predicted labels from Teams 1, 4, and 5. Similar figures for Team 2 and Team 3 are presented in Supplementary Fig. 1.

Across all three leading teams, microvascular breaks—manifested as discontinuities in small-caliber vessels—represent a dominant qualitative failure mode. These breaks are particularly pronounced in the cortical periphery of the kidney, where vessels are thinner, more tortuous, and more susceptible to partial volume effects (green arrows in Fig. 3). Although each team employed distinct architectural strategies, no one fully overcame this connectivity challenge, underscoring the intrinsic difficulty of preserving topology in multi-scale vascular networks.

For Team 1, the ensemble of two tailored 2.5D U-Net models with ConvNeXt-tiny encoders, combined with multiple loss functions including a custom marching cube loss, was designed to optimize boundary precision and NSD performance. The addition of an extra high-resolution stem and the use of 3D rotational augmentation during training enhanced small-vessel sensitivity. However, inference performed independently in orthogonal 2D planes—without rotational consistency—may have introduced slice-wise inconsistencies that propagated into three-dimensional disconnects, particularly for obliquely oriented microvessels. Thus, despite strong boundary optimization and high-resolution feature extraction, the predominantly 2D inference paradigm likely contributes to persistent microvascular fragmentation.

Team 4 combined 2D and 3D models, leveraging sliding-window 3D inference, TTA, pseudo-labeling, and BoundaryDOULoss. This hybrid design appears to provide stronger volumetric coherence compared to purely 2D ensembles, reflected in a lower prevalence of small isolated components within the kidney volume (yellow inset in Fig. 3). However, this architectural bias toward volumetric consistency and boundary agreement may favor stable detection of larger vessels at the expense of extremely fine peripheral branches, resulting in relative under-segmentation of the smaller microvessels. Importantly, Team 4’s post-processing step—masking the kidney boundary—effectively suppresses false positives outside the anatomical region, reducing spurious disconnected predictions (red inset in Fig. 3).

Team 5 ensembled three 2D U-Net models with boundary-weighted composite loss and pseudo-labeling on external data, aiming to sharpen vessel contours. While this approach improves edge definition, the reliance on 2D models without explicit 3D connectivity constraints likely contributes to the high degree of microvascular fragmentation observed. The model frequently detects portions of small vessels, but as short, disconnected segments rather than continuous structures. In addition, the use of soft pseudo-labels may propagate uncertainty into peripheral regions where vessel diameters can be as small as 1 voxel, potentially amplifying microvascular breaks.

A consistent cross-team observation is that predicted vessels are generally thinner than the gold standard labels, suggesting a systemic bias toward boundary sharpening and diameter under-estimation—likely influenced by boundary-based loss functions/metric and class imbalance mitigation strategies. Interestingly, Team 1 also predicts microvascular structures not present in the gold standard (orange arrowhead in Fig. 3). Upon inspection of the raw HiP-CT data, several of these structures appear to correspond to genuine vessels not captured during manual annotation. This highlights an important limitation of the gold standard itself: manual segmentation, even when performed by multiple expert curators, represents a high-quality reference but not a complete ground truth.

Overall, while architectural diversity (2.5D ensembles, hybrid 2D/3D models, boundary-focused losses, pseudo-labeling, and volumetric augmentations) led to high quantitative scores, qualitative inspection reveals that preserving microvascular connectivity remains a shared unresolved challenge, closely tied to the degree of explicit 3D spatial modeling and topological constraint within each approach.

**Fig. 4:**
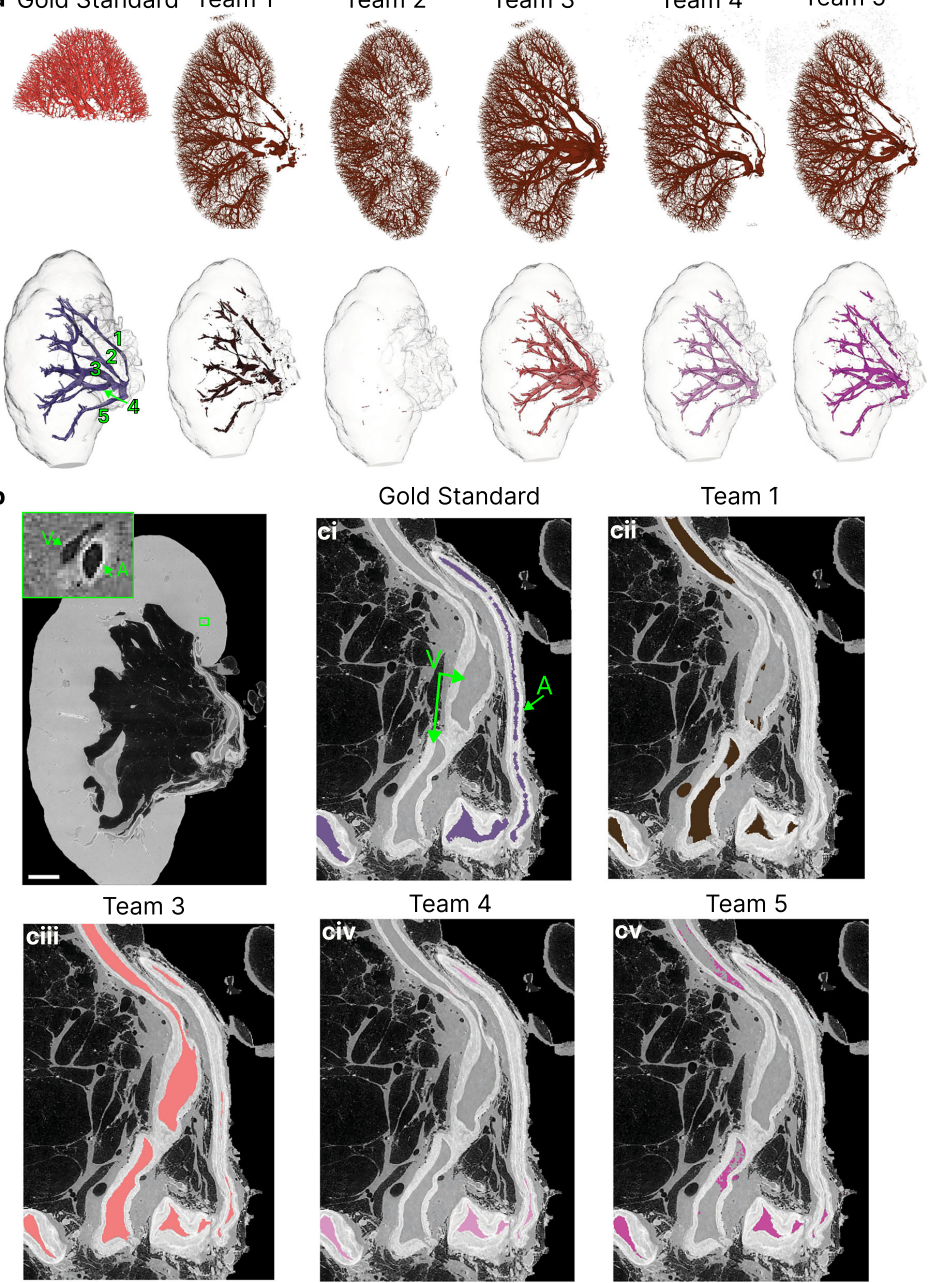
Qualitative analysis of the vascular trees. **a** Visualization of the 3D output for inference of each team on the whole intact kidney datasets for the private test data (Kidney 6). Top row shows the gold standard part of the whole kidney that was fully labeled and was part of the original competition dataset, along with the inference on the whole kidney for each team. The second row shows a 3D rendering of the large arteries (renal and segmental arteries) within the pelvis region of the kidney, the gold standard was manually segmented and the 5 segmental arteries are labeled. **b** 2D section through Kidney 6, green box and inset shows a smaller microvascular artery and vein where the distinction is far more visually apparent than for the larger vessels due to the absence of the thick wall in the vein. **ci**–**v** showing the segmentation of the large artery and vein for the GS and teams 1, 2, 3, 4, and 5. In general all solutions show poor distinction between the large artery and vein.

As the test datasets—both public and private—were only portions of the whole intact kidneys, we computed inference for each team’s model on the remaining parts of the private test set (kidney 6) (Fig. 4a) and the public test set (kidney 5) (Supplementary Fig. 4). The outputs in Fig. 4a serve to further demonstrate the differences between models, particularly in the ability to capture larger vessels which run through the hilum and medulla regions of the kidney. Team 1’s solution, which appeared to find many of the smaller vessels in test data, fails to identify the larger but collapsed arteries in the whole private test data (Kidney 6), and to a lesser extent in the public test data (Kidney 5). The larger vessels are evidently more fully captured by Teams 3–5 in both the public and private test datasets. The lack of generalizability in Team 2 is highly apparent when comparing the outputs for kidney 5 (Supplementary Fig. 4) and kidney 6 (Fig. 4a).

A secondary failure mode that is only apparent when viewing the larger vessel segmentation, is the failure of models to discriminate between the larger arteries and veins. Whilst in the microvessels this distinction is visually evident (Fig. 4b), in the large vessels both veins and arteries have thick walls and are easily mistaken (Fig. 4ci). Expert annotators are able to distinguish the two by tracing back either to the entry point into the kidney, where the renal vein and renal artery can be distinguished by anatomical location, or by tracing down to the smaller vessels where the visual difference becomes apparent. Across the 5 models, none were able to correctly segment the 5 segmental arteries of the kidney (Fig. 4a*second row*) with all demonstrating breaks where the vessels were collapsed or conflation of artery and vein. This is particularly evident when viewing Fig. 4ci–v ; Team 4 is the only solution that did not segment the larger veins which may be due to their volumetric consistency, however Team 3’s iterative pseudo-labeling is likely to be a cause for the oversegmentation of the large veins. It is also interesting to note that Team 1 demonstrates a surprisingly poor performance in the large vessels, which we suspect is largely due to the 2D inference mode discussed above.

### Morphological analysis of segmentations

While the quantitative segmentation metrics and qualitative inspection of the voxelized output provide insights into the performance of different solutions, a comparative morphological approach that aligns with the eventual intended use of the segmentations is highly relevant to assessing model biases and outputs. Given that our ultimate aim is to use the vascular segmentations to (1) model exosome transport, and (2) extract vessel morphology metrics to create a VCCF; the segmented vascular networks will be skeletonized. Skeletonization reduces the 3D voxel representation to a skeleton representation described by segments and nodes—with each segment defined by start and end nodes—and having a length, radius, and connectivity to other segments (see *Methods* for further details). Here, we skeletonize each of the predicted segmentation outputs to investigate how the differences in segmentation from the various models lead to variation in the extracted vascular network geometry.

**Fig. 5:**
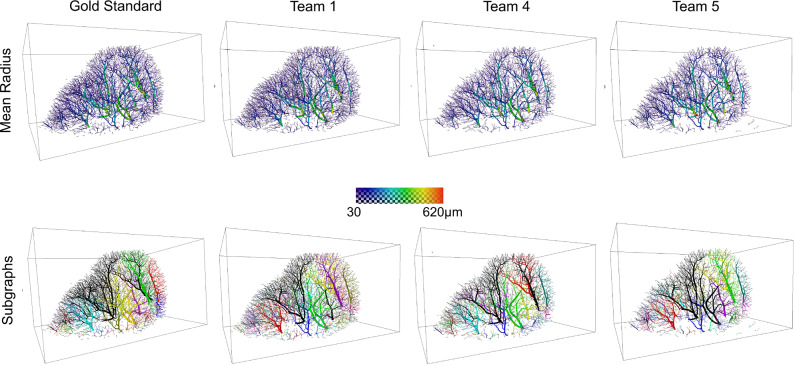
Skeleton visualization for morphological analysis. Visualization of the skeleton forms of the vessel network for the gold standard and teams 1, 4, and 5 for the Private test data (Kidney 6). In each case, each vessel segment is rendered with the mean radius. In the first row the vessels are colored according to each segment’s mean radius. In the second row each unconnected subgraph is in a different color.

Figure 5 shows the spatial variations in the mean radius and subgraphs (unconnected portions of the network) between model outputs. While the number of subgraphs varies between teams, Teams 1, 4, and 5 find the same larger vessel trees. Variations are mostly restricted to the smaller disconnected vessels at the kidney periphery which was also noted in the qualitative analysis. Similarly, while the spatial distribution of radii is similar, the absolute values vary between each solution and the gold standard. Supplementary Fig. 2 shows the same visualizations for Team 2 and Team 3. Supplementary Fig. 3 shows the skeleton forms of the vessel networks for all teams on public test data.

**Fig. 6:**
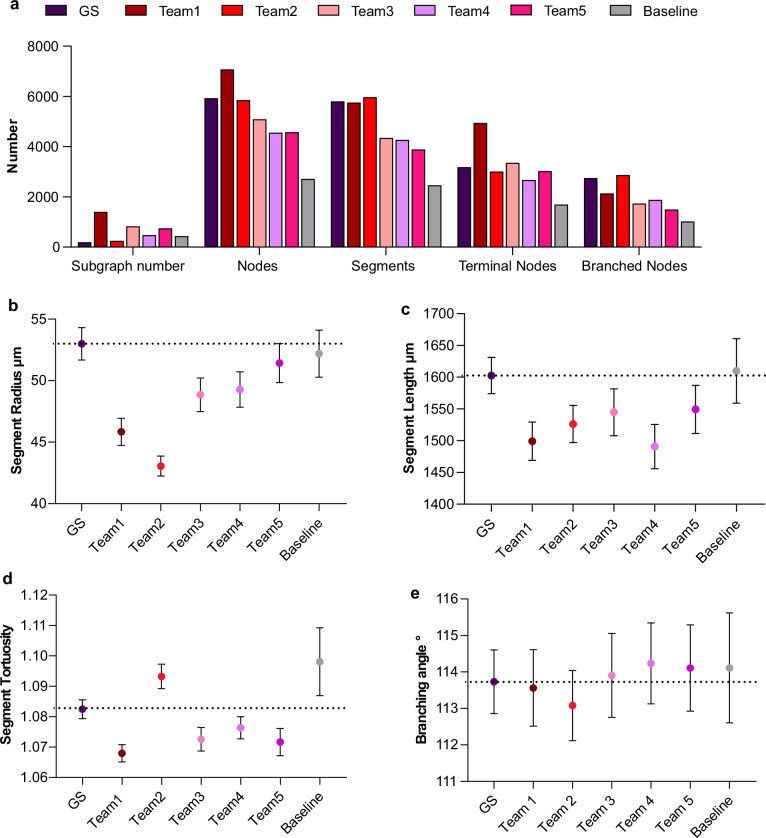
Vascular network geometry for morphological analysis. **a** Bar chart showing the number of subgraphs, nodes, segments, terminal nodes, and branched nodes for all team solutions as well as the baseline model predictions and gold standard (GS) labels for private test data. **b**–**e** Plots showing the radius, length, tortuosity of segments, and the branching angle between segments; mean and 95% Confidence Interval (CI) are shown for all metrics. Source data are provided as a Source Data file (see *Data Availability*).

Figure 6 highlights these morphological differences between the skeletonized networks geometry for the private test data (similar plots for public test data are provided in Supplementary Fig. 4). For all teams, the number of subgraphs is higher than the gold standard, which indicates many short unconnected segments that form the partial path of the vessels (Fig. 6a). The shorter mean length of segments (Fig. 6c) also supports this conclusion as does the proportions of terminal nodes compared to branched nodes which are higher than the gold standard for all teams other than Team 2. The mean radius (Fig. 6b) has large variability highlighting the challenge of accurately determining vessel thickness. In all cases, the mean radius is lower than in the gold standard, which appeared to be the case in the qualitative analysis. The thinner vessels are also reflected by the much higher NSD score where there is a 1-voxel tolerance (see Fig. 2). For tortuosity and branching angle, values closer to the gold standard and baseline models indicate better modeling of natural vessel shapes. As tortuosity is associated with length, it is interesting to note that while Team 4 has a lower vessel length than Teams 3 and 5, it has a higher tortuosity, indicating that many short but highly curved vessels are present in this case.

Team 1 has the highest number of segments, proportion of terminal nodes to branched nodes, and number of subgraphs, indicating the identification of many vessels but with a highly fragmented segmentation. Team 1 also has the second lowest mean radius indicating there is either an under segmentation of the vessel lumen or an increase in small vessels that may not be present in the gold standard.

Team 2 has the smallest number of subgraphs (247) comparable to that of the gold standard (199). It also has the most similar number of segments and proportion of terminal-to-branched nodes compared to the gold standard. This indicates better continuity in vessel segmentation, which can be attributed to the solution’s post-processing steps and is also reflected in the team’s relatively higher clDice score. Team 2’s lower radius and length might also suggest under-segmentation or more conservative thresholding.

Team 3 serves as the midpoint of the teams having the third highest number of subgraphs behind Team 1 and Team 5, with a similar proportion of terminal-to-branched nodes as Team 5. By comparison to Team 5, Team 3 has a lower mean radius and length suggesting a more conservative thresholding strategy leading to a thinner radius and slightly better predictions for smaller unconnected vessels.

Team 4’s lower vessel length coupled with the relatively higher mean radius, lower number of subgraphs, and lower proportion of terminal-to-branched nodes, indicates a model which is more targeted to the larger well-connected structures in the network rather than the smaller peripheral vessels, and reflects the masking of small false positive structures in the background outside the kidney.

Team 5 has the largest average radius and length, the third highest number of subgraphs, and the second highest proportion of terminal-to-branched nodes. This implies that while the vessel thickness may have been more accurately predicted (from radius and length), many small unconnected structures were still identified leading to the high subgraphs and high proportion of terminal nodes. The team’s lower position on the LB suggests these small unconnected structures were not smaller unconnected vessel fragments but likely came from noise predicted outside the kidney, as seen in Fig. 3 and Fig. 5.

Morphological analysis based on the skeletonization of segmented vascular networks provides an additional and alternative comparison between segmentations. Such morphological characterizations are one of the most common downstream analyses performed on these types of segmentations as they facilitate statistical comparison between networks to test biological hypotheses and are a necessary first step for multi-scale flow modeling^4^. It has been shown that even relatively small differences in measures such as Dice or NSD can lead to large differences in skeletonization outputs and consequently into statistical comparisons between networks or outputs of flow modeling^20^. From the above analysis we can conclude that even though the top-5 networks achieve relatively close scores in NSD, the skeletonized networks they produce have a very high variability, and would lead to very different simulated flow properties. In particular, the connectivity of the networks as highlighted by the subgraph number and the relative number of branched and terminal nodes highlights the challenge with segmenting well-connected networks. This type of analysis can be used to drive future networks by directing post-processing to correct for or prioritize connectivity as a feature of vascular segmentation models. Supplementary Fig. 6 shows the morphological analysis results on the additional dataset.

## Discussion

Vasculature segmentation is a highly complex and challenging task in medical image segmentation due to the low proportion of vessel voxels, complex structure, and variable nature of human vasculature across different individuals. Citizen science enables experts and hobbyists alike from industry, academia, and government globally to engage in open and collaborative experimentation and development of solutions. Furthermore, all data and code are openly shared, serving as a template for future algorithm performance evaluations and comparisons for HiP-CT data.

Citizen science competition platforms such as Kaggle are ideal for bringing more awareness about niche scientific problems to a wider scientific and engineering community–especially in data science and AI domains–beyond the scientific journals. Posing problems for community-driven experimentation and solutions has both advantages and disadvantages. In terms of advantages, it provides a way to bring together domain experts–scientists and engineers–from across the world that have experience with machine learning and imaging problems, including biology-focused problems. The participants are focused on solving a particular problem and are able to do that in collaboration with other participants by actively forming teams or sharing their code and findings in the public forums. Imaging and domain experts also benefit from such competition paradigms as they are forced to think about their problems as well as biology-appropriate metrics concisely and clearly for the wider community.

In terms of disadvantages, there are dataset size, time, and resource constraints for the participants, and the solutions they devise reflect that. Additionally, the gamification of the leaderboard can lead to over-optimization of solutions on singular competition metrics. Overall, the competition format expedites experimentation of numerous machine learning models, hyperparameters, and pre- and post-processing techniques, providing a set of fewer high performing solutions for the specific task and dataset which can then be used by the wider scientific community to benefit their own approaches.

The competition revealed three interconnected methodological themes representing advances in applying deep learning to imaging modalities like HiP-CT with limited training data. Firstly, pseudo-labeling strategies evolved from basic external dataset labeling to sophisticated iterative refinement exploiting distributional similarity between datasets, demonstrating that effectiveness depends on understanding relationships between labeled, unlabeled, and target data—applicable to any domain with expensive annotation but abundant unlabeled data. Secondly, vascular segmentation requires alignment between boundary- and topology-focused optimization, both in terms of metrics and loss functions, emphasizing the importance of approaches that collectively optimize for vessel volume, boundary, and connectivity. Lastly, diverse architectural strategies employed by different teams illuminate trade-offs between spatial (2D/3D) context, patch size, and computational cost, with 2.5D or multi-axis 2D approaches enabling larger patch sizes than pure 3D approaches while retaining limited volumetric context—an architectural strategy potentially generalizable to other high-resolution 3D modalities where computational constraints may limit full 3D processing.

While this competition has generated interesting solutions for a fairly new imaging modality, several known limitations persist: (1) the models were trained on a relatively small dataset, which increases the risk of overfitting; (2) segmenting both the very fine and the large microvascular network accurately still remains a challenge; (3) the competition might rely on specific evaluation metrics that do not fully capture the clinical relevance or quality of the segmentation, potentially overlooking important aspects of model performance in real-world applications; and (4) some models are computationally expensive and might be impractical or inefficient for deployment at scale.

Testing the inference of the top-five winning solutions on additional high resolution VOI highlights limitations further, specifically in terms of performance and skeletonization. While two of the five models perform favorably on the additional dataset by generalizing to a different kidney dataset (in terms of imaging set-up, resolution, and size), it is clear that more training datasets would be needed at higher resolutions for improving the performance further. That being said, such models would be beneficial in a human-in-the-loop setting to iteratively improve model performance and segment datasets faster.

In the context of HiP-CT vascular segmentation, biomedical interpretability and utility are best understood through the reliability of downstream morphological quantification and flow modeling rather than visual plausibility alone^40^. Skeletonization enables construction of graph-based representations of the vascular tree, from which branching topology, vessel hierarchy, resistance distribution, and simulated flow dynamics can be computed. These analyses underpin modeling of blood flow, drug delivery, and exosome transport, providing clinically and biologically meaningful insight beyond conventional segmentation metrics.

Most skeletonization methods are not designed for multi-scale vascular networks such as those captured with HiP-CT, making parameter selection challenging when attempting to preserve both large trunks and fine microvasculature. For example, parameter choices that allow accurate capture of the smallest vessels often introduce spurious sub-branches along large vessels, whereas more restrictive settings risk suppressing peripheral structures. As a result, small segmentation inconsistencies can propagate disproportionately into skeleton-based metrics and graph representations, complicating downstream analysis and interpretation.

Two properties are therefore essential for biomedical utility: preservation of vascular connectivity and accurate delineation of vessel boundaries, particularly in large-caliber vessels that dominate hemodynamic resistance. While errors in large vessel segmentation–a feature of the top five models–are comparatively easier to detect and manually correct, microvascular connectivity errors are more difficult to identify and rectify, and thus pose a greater challenge for automated pipelines. Morphological analysis revealed that most teams produced a high number of unconnected components and a reduced proportion of branched-to-terminal nodes relative to the gold standard. Such fragmentation directly limits flow modeling: in network-based simulations, each terminal node requires assignment of a boundary condition, and an inflated number of disconnected terminals increases model sensitivity to imposed boundary conditions rather than intrinsic network structure^20^. Consequently, connectivity errors do not remain local but propagate into substantial differences in predicted functional behavior.

The recurrent presence of microvascular breaks across models therefore represents not merely a segmentation imperfection, but a key barrier to robust physiological modeling. Post-processing strategies that remove isolated components, such as those applied by Team 2, or more advanced graph-based reconnection approaches, would constitute important next steps toward enabling reliable flow-based analyses from automated HiP-CT segmentations.

In future, the authors would like to further generalize the winning solutions and train on other 3D HiP-CT datasets to explore the vasculature trees, exosome flow patterns, and morphological differences in organs other than the kidney. The authors expect the solutions, metrics, and datasets provided in this work will benefit the 3D vascular segmentation community and particularly the X-ray phase-contrast and HiP-CT communities for vascular segmentation in other organs beyond the kidney.

## Methods

All kidneys were collected from deceased donors following local ethical regulations and approvals. All kidneys, except Kidney 2, were collected from donors who had consented to body donation to the Laboratoire d’Anatomie des Alpes Françaises prior to death. Kidney 2 was obtained during a clinical autopsy at the Institute of Pathology in Wuppertal with consent from the next of kin; the ethics vote is registered in Hannover under the ethics vote number BO_K_2021. The transportation and imaging protocols received approval from the French Health Ministry. All raw imaging datasets have been previously published on the Human Organ Atlas portal (see Supplementary Table 1).

### Kaggle platform

The private leaderboard for identifying the five winners was finalized on February 8, 2024. The teams submitted their inference code, after training their ML models, on the Submission portal. The submitted code was then run over the public test set to rank the teams on the public leaderboard. Teams typically use this score to validate their models. With each submission, the code is also run on the private test set for preliminary rankings on the private leaderboard (not visible to teams). Teams can make an unlimited number of submissions before the competition deadline but are limited to five submissions per day. On competition end, the teams can choose up to two solutions to submit as their final submissions, which are then scored on the private test set to rank the teams on the final private leaderboard. All scoring is done using the NSD as the evaluation metric and the top-5 teams on the final private leaderboard are selected as winners. The competition website is available at https://www.kaggle.com/c/blood-vessel-segmentation.

### Hierarchical phase-contrast tomography data acquisition

HiP-CT is a propagation phase-contrast local tomography technique, as described by Walsh et al.^2^. Data acquisition follows the sample preparation and scanning protocols outlined in prior work^2,41^. Briefly, organs are fixed, partially dehydrated, and stabilized for tomographic scanning using an agar-ethanol mixture. HiP-CT scans are performed on one of two beamlines, BM05 or BM18 at the ESRF. The scans are conducted hierarchically; the entire organ is first scanned at ca. 25 µm per voxel, followed by local tomography at specified locations within the intact organ at ca. 6.5 µm per voxel and ca. 1.3–2.5 µm per voxel.

Tomography scans are reconstructed into image volumes using a filtered back projection algorithm as detailed in prior work^41^. The final volumes consist of isotropic 3D image datasets, where higher resolution volumes of interest are registered within the larger organ volume using a rigid transformation. The contrast within these images arises from interference patterns caused by refraction of X-rays as they pass through the samples. Refraction is caused by physical density differences within the sample, and thus the modality particularly highlights edges between tissues with different densities.

#### Gold standard label acquisition

Five human kidneys were used to create the competition dataset. Three kidneys (Kidney 1^42,43^, Kidney 2^44^, and Kidney 3^45^) were used for the training set. Two kidneys (Kidney 5^46^ and Kidney 6^47^) were used for the test set—Kidney 5 as the public test set and Kidney 6 as the private test set. The basic scan parameters and demographic information of subjects can be found in Supplementary Table 1. The segmentation of the five kidneys was conducted using Amira Version 2021.1. Initially, the raw image data underwent average binning, either x2 or x4. In x2 binning, the resolution was reduced from approximately 25 µm to 50 µm voxel size. In x4 binning, the resolution was reduced from 15.8 µm to 63.1 µm voxel size.

A 3D median filter was then applied using Amira-Avizo v2021.1 with 3 iterations and a 26-voxel neighborhood. To enhance the appearance of vessels, background detection correction was performed (Amira v2021.1; default settings). The segmentation process involved semi-manual techniques using Amira v2021.1’s magic wand tool, an interactive 3D region-growing tool. Curators selected a seed voxel within a vessel slice and refined parameters such as intensity threshold, contrast threshold, and hard-drawn limits to determine the stopping criteria for the 3D region growing. In cases where vessels were infilled with blood or largely collapsed, a manual voxel paintbrush tool was used to correct or fill the vessel in every slice. Further details and supplementary video of the semi-automated segmentation protocol is outlined in prior work^3^. To ensure segmentation quality and provide error estimates on the gold standard, an expert segmentation validation process was implemented. An initial curator applied the above procedure on a dataset using three orthogonal views. An independent curator re-labeled the data adding to the labels of the initial curator, again using the three orthogonal planes. A third curator (referred to as proof-reader) was presented with 5–7 randomized 2D sections of the data in any one of the three orthogonal planes. They counted the number of vessel cross-sections in the slice. They recorded the number of true positive and false negative vessel cross-sections that were segmented. Note that a true positive means that the curators 1 and 2 have visually correctly labeled the vessel but does not assess the quality of the segmentation on a pixel-by-pixel basis. This ratio of true positive and false negative is used as the estimate for the number of vessels correctly segmented. The proof-reader then returns the data to the two curators, with the estimated segmentation proportion for each 2D area. The curators 1 and 2 then focus on 3D segmentation in areas where the proof-reading has highlighted the lowest vessel segmentation proportion. The process repeats iteratively until the proof-reader does not find any false negatives^3^. Note that false positives are not considered in the labeling/proofreading process as these are easily detected owing to their lack of connectivity to the main vessel tree. All data is made publicly available, see *Data Availability*.

#### Additional dataset

The additional dataset provided is a Volume of Interest (VOI) from within the right kidney^48^ of donor 1. It was imaged at 6.5 µm/voxel (binned to 13 µm/voxel) on BM05 using the set-up and parameters in the metadata (see DOI in Supplementary Table 1). This dataset, while from the same kidney as other datasets in the training data, still represents a challenge for training segmentation models. HiP-CT imaging set-ups are bespoke to each sample and each resolution. Aside from the clear difference in pixel size, and thus the relative size of features, there are also many subtle differences in the imaging set-up, for example, the X-ray optic used for this imaging set-up utilizes a different lens and filters and thus, the energy profiles of the x-ray beam itself are different. Consequently, while the images may appear to the human eye as simple upsampled or downsampled versions of the higher and lower resolutions, the image statistics of the volume and the relative contribution of X-ray phase and X-ray attenuation result in the dataset having different image intensity and relative contrast for structures compared to the other datasets in the training data. This is a fair and realistic challenge for such models as there are ever increasing numbers of HiP-CT kidney datasets being made openly available on the Human Organ Atlas (human-organ-atlas.ersf.eu) that will need this type of generalization capability to make them easily usable by the community for large scale analyses.

### Evaluation metrics

The submitted solutions were ranked in the competition using NSD with tolerance (t) set to 0. NSD determines what fraction of segmentation boundary is predicted correctly. A boundary element is considered correctly predicted if the closest distance to the reference boundary is smaller than or equal to the specified threshold (tolerance). The tolerance determines the acceptable amount of deviation in pixels. The value of NSD lies between 0 and 1. The specific implementation^23,24^ proposed by Google DeepMind is used in the competition with an optimized version available as a notebook on the Kaggle platform at https://www.kaggle.com/code/metric/surface-dice-metric. To further evaluate the winning solutions and their performance post competition, four auxiliary metrics were chosen based on prior work by the authors^5^: Dice Similarity Coefficient (Dice)^49,50^, Centerline Dice (clDice; https://github.com/jocpae/clDice)^51^, ASSD^24,52^, and NSD (with tolerance = 1). ASSD was computed using the MONAI library^53^ v1.3.0.

### Morphological analysis code

Vasculature in segmentation masks is reduced to spatial graph representations of the network through skeletonization. The resulting spatial graph describes the vessel network in terms of four entities: nodes, points, segments, and sub-segments. A segment is defined as being between a start node i and end node j; which correspond to either a branching point leading into another segment branch or a terminal end where no further branches were detectable. Nodes have an ID and a 3D spatial position (x, y, z). Between the start node i and terminal node j of each segment lie sub-segments with points, marking the start and end of each sub-segment able to capture the curvature of the segment. Each sub-segment has an associated radius *R*_s_ and length *L*_s_.

To create a spatial graph from a segmented image, we utilize an implementation of the parallelized version of the distanced ordered medial thinning algorithm^20,54^ in Amira v2021.2 termed the Autoskeleton plugin. The radius of each subsegment is estimated using 1/5th of the maximum Chamfer distance and the parameters applied are smooth = 0.5, attach_to_data = 0.25, and iterations = 10. We extract morphological parameters from the spatial graph following the definitions from prior work^20^. See *Code Availability*.

### Participation analysis

At the conclusion of the competition, participation metadata becomes publicly available on Meta Kaggle^55^—Kaggle’s public data on competitions, users, submission scores and kernels. This data is used to understand how the competition unfolded over its 3-month period. Analysis is implemented using standard python packages for data science such as Pandas, NumPy, Matplotlib, and Seaborn; creating all visualizations in Jupyter Notebooks, see *Code availability*.

### Statistical analysis

Kendall’s Tau^37–39^ (also called Kendall’s Rank Correlation) is used to quantify the agreement between two rankings and is independent of the number of entities ranked. Tau values closer to 1 mean a strong positive correlation between the two rankings—value of 1 means perfect alignment— whereas values closer to -1 mean a strong disagreement. A *p*-value associated with the tau value indicates the statistical significance of the correlation; lower *p*-values (closer to 0) indicate higher significance of the relationship between the two rankings such that it is unlikely to occur by chance. Kendall’s tau is computed using the implementation in the Python Scipy^56^ library.

### Team 1 solution details

#### Summary

Team 1 proposed a customized U-Net^25^ based architecture model using a lightweight encoder named ConvNeXt-tiny^27^ with additional stem blocks^57^ to improve the feature extraction. A data augmentation technique, including random 3d slice rotation, was used to tackle the challenges related to the data. To make sure that the proposed model is optimized well, they trained the model with an ensemble loss combining focal loss^58^, dice loss^33^, boundary loss^34^ and a customized marching cube loss. The main goal of marching cube loss is to directly optimize the surface dice. The data were processed across three axes with large inference size and test time augmentation for inference.

#### Data preparation

They trained the proposed model using all available data, including high-resolution and sparsely labeled data. Then, all slices were randomly resized to size 1536 × 1536. To increase the size of the training data, they sliced the whole volume along different axes and views. Heavy data augmentation, including flipping, scaling, adding noise, and intensity modifications, was utilized. Moreover, they randomly rotated the sampled slice in 3D space as online data augmentation. Complete data augmentation recipe is available at https://github.com/cns-iu/hra-sennet-hoa-kaggle-2024/blob/main/winning-team-solutions/team-1/training_code/dataset.py.

#### Model

The general architecture of the model includes a 2.5D U-Net^25^ based architecture stacking 3 consecutive slices on channel dimension as input for the model to leverage 3D information. The encoder part of the model includes the ConvNeXt-tiny model^27^ combined with an extra stem block^57^ to extract high-resolution features. The decoder part of the model is a standard U-Net decoder.

The final submission involved an ensemble of two 2.5D U-Net ConvNeXt-tiny models, one with 3D rotation and one without 3D rotation. The team found that standalone 2.5D models exhibited inferior performance compared to their 2D counterparts, however with the integration of custom marching cube loss and 3D random rotation techniques, the performance of the 2.5D models demonstrated significant improvement. The team created an efficient implementation of 3D image rotation for faster on-the-fly data augmentation, with the code available at https://github.com/cns-iu/hra-sennet-hoa-kaggle-2024/blob/main/winning-team-solutions/team-1/training_code/rotate_slice.ipynb.

For the loss function, this team utilized a *combination of four losses* including focal loss^58^, dice loss^33^, boundary loss^34^, and a customized Marching Cube Loss.

#### Customized Marching Cube Loss (MCL)

Since the competition uses the NSD at *tolerance* = *0*, the metric can be simplified as weighted cube dice:

$${surface}\,{dice}=\,\frac{{\sum }_{{tp}}\,\,{S}_{{pred}}\,+\,{\sum }_{{tp}}\,\,{S}_{{gt}}}{{\sum }_{{all}}\,\,{S}_{{pred}}\,+\,{\sum }_{{all}}\,\,{S}_{{gt}}}$$

If the prediction and the ground truth mask cube both have non-zero surface area, this cube is true positive. The weight *S* for different surface cubes is marching cube surface area. A cube is composed of 8 voxels, each voxel has value 0 or 1.

Replacing the binary mask with probabilities and the surface area with mean surface area expectation defines a soft surface dice. *p* denotes the surface probability:

$${soft}\,{surface}\,{dice}=\,\frac{{\sum }_{i}\,\,{p}_{i}{\hat{p}}_{i}\,({w}_{i}\,+\,{\hat{w}}_{i})}{{\sum }_{i}\,\,{p}_{i}{w}_{i}\,+\,{\sum }_{i}\,\,{\hat{p}}_{i}\,{\hat{w}}_{i}}$$

$${w}_{i}=E[{area}]\,=\,{\sum }_{j=1}^{256}\,{p}_{i,j}\,.\,{{area}}_{j}\,$$

There are 256 patterns for a cube, and each of them defines a surface known as a marching cube. So, we can convert the output voxel probability into cube probability by 256-channel 3D convolution.

But 256-channel 3D convolution increases computation and memory overhead, and because the cube distribution only influences the surface area weight, we divided 256 patterns into 3 classes: foreground, background, and surface.

Use 2-channel convolution to get foreground and background probability, and the following equation to get surface probability:

$$p({surface})=1\,-\,p({background})\,-p({background})$$

$${where;}\,p({cube})=p({v}_{1},{v}_{2},{v}_{3},...,{v}_{8})\,=\,\exp ({kernel}*\log p(v))$$

Then, replacing the surface area expectation with the ground truth surface area:

$${MCL}=\,1-\frac{2\,{\sum }_{i}\,\,{p}_{i}{y}_{i}{w}_{i}}{{\sum }_{i}\,\,{p}_{i}{w}_{i}\,+\,{\sum }_{i}\,\,{y}_{i}{w}_{i}}$$

In practice, the surface area weight has minimal influence on the final performance of the mentioned loss. So, the loss mentioned above only considers the surface cubes (ground truth foreground and background cube’s surface areas are zero). Therefore, ignoring the surface area weight, the team used the mean of foreground, background and surface dice losses as customized Marching Cube loss:

$${MCL}=1-\frac{1}{\left|K\right|}{\sum }_{k\epsilon K}\,\frac{2{\sum }_{i}\,\,{p}_{i}^{k}{g}_{i}^{k}}{{\sum }_{i}\,{\,p}_{i}^{k}+{\sum }_{i}\,\,{g}_{i}^{k}}$$

$$K=\{\,{foreground},\,{background},\,{surface}\}\,$$

The code implementation of the total loss is given at: https://github.com/cns-iu/hra-sennet-hoa-kaggle-2024/blob/main/winning-team-solutions/team-1/training_code/loss.py (see ***line 66*** for custom marching cube loss implementation).

#### Training and inference details

This team trained the proposed model using a 32 batch size and a gradient accumulation process^59^. AdamW^60^ with additional WarmUpConsineAnnealing^61^ was used to optimize the model. The model was trained for 30 epochs.

In the testing process, all the slices were converted and resized to 3072 × 3072. The torch compile algorithm^59^ was utilized to accelerate the inference process. Moreover, the data were processed across three axes with large inference sizes and test time augmentation.

### Team 2 solution details

#### Summary

Team 2 used a customized 3D U-Net^26^ model for segmenting the kidney vessels. To overcome the generalizability problem, data augmentation techniques, including random positions and rotations, were used. Eventually, morphological post-processing techniques were used to remove small false positives and improve the model performance.

#### Model

The general architecture of the proposed neural network is based on 3D U-Net^26^. However, to overcome the problem of gradient vanishing, simple convolution layers were replaced by ResNet blocks^57^ in the encoder part of the model. To decrease the computational cost, all traditional up-convolutional layers have been replaced by up-sampling operations.

Online data augmentation techniques, specifically affine transformations such as rotation, scaling, and shearing, were employed to prevent overfitting. This process yielded a substantial increase in the number of patches, reaching over 7k for every epoch.

The *key insight* from this team’s approach is the post processing: After predicting the vessels, a connected component analysis over the predictions is done using depth-first search algorithm^62^ to identify the smaller unconnected, distantly placed chunks and remove them to increase the model’s overall performance. The code implementation for this step is available at https://github.com/cns-iu/hra-sennet-hoa-kaggle-2024/blob/8c03024c8302f9bfe1fc8404eac0add307c146d2/winning-team-solutions/team-2/blood-vessel-segmentation/src/submit.py#L102.

#### Training and inference details

This team trained the proposed model with a batch size of 4 and a size of 128 × 128 × 32. For optimization, they used Focal loss^58^ and cosine decay scheduling^61^. The training lasted 2 weeks, and the maximum number of epochs was 700. 0.25 was used as the ultimate threshold for the inference to extract binary masks for vessels.

### Team 3 solution details

#### Summary

Team 3 employed a multifaceted approach to tackle the competition. They used a two-step process to refine sparse labels using dense labels from part of the training dataset. Initially, they trained two different UNet architectures (maxViT512^28^ and EfficientNetv2^63^) on densely labeled images to generate supplemental labels for sparsely labeled ones. These models were then further trained using the enhanced datasets, which included both dense and newly generated pseudo labels. The team trained three UNet models: EfficientNetv2, SeResNext101^64^, and MaxViT512, and one UNet++^65^ using all real labels from all kidneys plus pseudo labels.

#### Model

The main model, MaxVit512, represents a sophisticated UNet-based architecture combining CNN and Transformer methodologies, tailored specifically for medical image segmentation. It utilizes a hybrid multi-axis vision transformer mechanism, where both convolutional and self-attention mechanisms are employed at each stage of the decoder. This approach significantly enhances the model’s ability to distinguish between target objects and the background, crucial for effective segmentation. The MaxVit512 architecture, with its strategic use of a hybrid decoder, ensures high efficiency in segmentation with a reasonable computational and memory footprint. The models typically operate with a batch size of 32, allowing efficient processing of large datasets without compromising performance.

#### Training and inference details

Recognizing the variation in magnification between training and test sets (50 µm/voxel for training and public test sets, and 63 µm/voxel for the private test set), the team adjusted their training strategy to simulate the lower resolution of the private test set by scaling images down. They applied a ShiftScaleRotate augmentation, which randomly shifts, scales, and rotates the images to enhance model robustness to variations in image presentation. Training and inference were performed *along each axis* (x, y, and z) to manage varied resolutions and sizes, using solely 2D models. They maintained a consistent model resolution, opting for 512px for most tasks but switching to higher resolutions when supported by the dataset to minimize potential accuracy loss in smaller resolution data.

The team trained models on all available data once they observed stable convergence, maximizing learning from limited datasets. They used dynamic threshold values to maintain stability in model predictions, crucial for consistent segmentation performance. Given the intensity variations across different datasets, they implemented heavy intensity augmentation strategies to enhance model robustness. For final submissions, the team used both a single model and an ensemble of the four models mentioned, with both approaches yielding the same score of 0.727. Their script utilized CUDA for GPU acceleration, ensuring fast prediction processing. Through these strategic maneuvers, the team effectively managed dataset variabilities and optimized their models for high performance. Overall, MaxVit512 scored 0.727, and the ensemble submission also scored 0.727.

#### Refining pseudo labels from sparse to dense iteratively

Since two training datasets (*kidney 2* and *kidney 3 sparse, see* Table 1) were sparsely labeled, the team decided to generate pseudo labels for the remaining unlabeled vessels in these datasets, and then further use them for training the complete model. The key was to not generate pseudo labels on both datasets simultaneously but instead use an iterative approach. Since kidney 3 sparse should have close distribution to kidney 3 dense, models trained on kidney 3 dense should perform well on kidney 3 sparse. Hence, the process refined kidney 3 sparse first:

1. Train UNet (MaxVIT512) and UNet (EfficientNetv2s) using kidney 1 and kidney 3 dense.
2. Generate pseudo labels for kidney 3 sparse using the models trained in step 1.
3. Resume the training of the models for a few more epochs using kidney 1, kidney 3 dense, and kidney 3 sparse (pseudo labeled).
4. Generate pseudo labels for kidney 2 using the models trained in step 3.
5. Train 3 UNet models (MaxVIT512, EfficientNetv2s, and SeResNext101) and 1 UNet++ model on all kidney datasets, including pseudo labels.

To generate hard binary labels from pseudo labels in step 2 and 4, the process was defined as:

1. Run inference on the sparse kidney dataset, save the mask in float format instead of binarizing them at this stage.
2. Calculate the number of positive pixels.
3. Calculate True Positive (TP), False Positive (FP), and False Negative (FN) at different thresholds, and select the threshold that is closest to the label density originally provided (65% for kidney 2 and 85% for kidney 3 sparse).

### Team 4 solution details

#### Summary

The fourth team’s final model strategy combined 2D and 3D models, utilizing d4 TTA^66^ to improve segmentation results. They applied a multi-view TTA approach to the 2D models and conducted training using a 2-fold setup, selecting kidney_2 and kidney_3_dense as validation sets. They ensembled the models by assigning equal weights to both 2D and 3D models. Their training datasets included kidney_1_dense, kidney_2, kidney_3_dense, kidney_3_sparse, and pseudo labels. The team transitioned from slice-wise normalization to stack-wise normalization using percentiles to optimize model learning.

#### Model

The team’s main models included EfficientNet family models with a UnetPlusPlus decoder and SCSE attention^67^, integrated from the segmentation_models_pytorch library. EfficientNet-B5 was particularly effective for feature extraction, capturing complex patterns in images. Unet++ enhanced feature propagation, crucial for accurately delineating boundaries and maintaining spatial details during encoding and decoding. Despite testing various architectures, the EfficientNet-B5-UNet++ model demonstrated superior performance. Their 3D model was based on the nnU-Net architecture but used the DynUnet from the MONAI library, with training over 500 epochs using SGD. Both the 2D and the 3D model used RAdam optimizer with cosine annealing learning rate schedule, and BoundaryDOULoss (the 3D model used the 3D version).

#### Training and inference details

Training involved a multiview setup, stacking images in tensors and slicing along different axes. They employed a weighted sampling approach based on sample sparsity, with denser samples given a standard weight and sparser samples adjusted accordingly. For example, kidney_1_dense had a weight of 1, while kidney_2 was assigned a weight of 0.65. Dynamic augmentations like CutMix^68^ (applied with a 0.5 probability), shifts, flips, and brightness adjustments were crucial for model robustness. The 3D model’s augmentation strategy was simpler, involving d4 augmentations and random crops, with cropping adjusted to 192 × 192 × 192 to focus on volumetric capture. For CutMix augmentation, only slices from the same view could be mixed.

Pseudo labeling was done using an ensemble of 2D models with data from https://human-organ-atlas.esrf.eu/, excluding some data to prevent leakage. Post-processing techniques, such as multiplying 3D model predictions with 2D model-derived ROI masks, significantly enhanced predictions. Ensembling predictions from 2D and 3D models with equal weighting further boosted performance. The team found that incorporating BoundaryDOULoss^35^ early in the competition significantly improved model performance, with a +2% increase in cross-validation metrics, +1.5% on the public leaderboard, and +5% on the private leaderboard.

### Team 5 solution details

#### Summary

Team 5 solution applied a 2D segmentation model using HiP-CT slices as inputs. The final predictions were given by ensembling three 2D U-Net models trained on *xy*, *yz* and *xz* axes, respectively. The team trained the model on kidney_1, then kidney_2, and validated on kidney_3. To achieve better results, they applied several technical implementations such as pseudo-labeling from additional data, boundary loss and spatial resolution interpolation.

#### Pseudo-labeling

Considering that data is important to train an effective deep neural network, the team involved two additional datasets: LADAF-2020-31 kidney^69^ and LADAF-2020-27 spleen^70^, from the Human Organ Atlas (https://human-organ-atlas.esrf.eu/) into the training. A 4-step training scheme was applied to gradually generate pseudo labels for the new datasets and incorporate them into training:

1. The team trained a base model on kidney_1 and pseudo-labeled, kidney_2.
2. Kidney_2 was incorporated into the training data together with kidney_1 to re-train the model. Then, they used this model to pseudo-labeled the LADAF-2020-31 kidney.
3. Similarly, they pseudo-labeled LADAF-2020-27 spleen dataset from the model re-trained on kidney_1, kidney_2 and LADAF-2020-31 kidney.
4. Finally, the model was trained on four datasets.

The 4-step training scheme enabled generating pseudo-labels for the new dataset. However, pseudo-labels can introduce training noise with false positives which are difficult to completely remove in this scheme without post-processing. To overcome this problem, the team used soft labels, the probabilities after sigmoid function, as pseudo-labels instead of hard labels.

#### Loss

As surface dice is the metric in this competition, evaluating the predicted boundaries of the vasculature, the team applied a boundary weighted binary cross-entropy loss as equation:

$$L=-{\sum }_{i=0}^{i=n-1}\,(1+\alpha {boun}{d}_{i})\left[{y}_{i\,}{logp}({y}_{i})-(1-{y}_{i})\log (1-p({y}_{i}))\right]\,,$$

$${boun}{d}_{i\,}=0\,{if}\,{y}_{i}\,{is}\,{not}\,{on}\,{boundary},\,1\,{if}\,{y}_{i}\,{is}\,{on}\,{the}\,{boundary}$$

where n is the size of a mini-batch. α is a boundary weight and ${boun}{d}_{i}$ is a binary value, indicating if the value ${y}_{{i}}$ is on the boundary. The last term is a typical binary cross-entropy function. The team set α as 0.9 to roughly double the loss on the boundary pixels. Apart from a boundary weighted binary cross-entropy loss, the overall loss function added dice loss^33^ and focal loss^58^.

#### Models

Four different 2D backbone networks: effnet_v2_s, effnet_v2_m, maxvit_base and dpn68 were ensembled for the final submission. They are implemented using a python package—Segmentation Models PyTorch (SMP)^71^. The input was cropped from *xy*, *xz* and *yz* axes in size of 512 × 512, followed by pre-processing methods, such as 2D rotation, intensity contrast changes, and horizontal and vertical flipping. After training, the inference was done with a sliding window method with an overlap size of half the input size. A key feature was to do model ensembling at the logit level rather than at the probability level.

#### Inference

Team 5 solution highlighted that 3D interpolation to make the voxel sizes of the training data and test data the same is important when performing vasculature segmentation inference on HiP-CT data. In this competition, the training data of kidney 1 to kidney 3 are ~50 µm/voxel, however, the test dataset of kidney 6 is ~63 µm/voxel. Team 5 applied a rescaling operation through 3 axes even though training was performed on 2D slices. The rescaling was implemented by trilinear interpolation directly on the 3D image (not individual 2D slices) as part of post-processing on final predictions.

### Useful strategies from other teams

#### Pre-processing

The kidneys were imaged by HiP-CT which requires the X-ray beam and scan setup to be independently tuned for every individual sample. Thus, scan parameters and hence the ranges of voxel intensities vary often dramatically between samples. This effect introduces weight bias and shifts when training deep neural networks. Therefore, several approaches were applied in this competition to construct a uniform input intensity space for stable training. Popular pre-processing techniques were normalization with global maximum and minimum across three training kidneys and intensity augmentation in training and validation. However, for 2D models, normalization of individual slices performs better than normalization with global maximum and minimum from 3D volume (https://www.kaggle.com/competitions/blood-vessel-segmentation/discussion/469022).

#### Model selection

HiP-CT can image intact human organs, with isotropic voxels enabling clear visualization of 3D structures in any orientation. This inherently inspires the application of 3D as well as 2.5D and 2D segmentation models. Therefore, many teams explored different models for this task, reporting that 2D models were more efficient for achieving better results, due to the challenges of setting the hyper-parameters for 3D models. Due to limitations in computational resources for many competitors, 3D model input patches were smaller (input dimensions = $64\times 64\times 64$) and thus contained less spatial context, compared to popular input settings of 512×512 in 2D and 2.5D models. If the input dimension were increased in 3D models, the batch size had to be reduced.

#### Post-processing

For HiP-CT scanning, organs are physically stabilized in cylindrical jars using an ethanol–agar or formalin-agar gel, so the images also contain the irrelevant background areas of the surrounding ethanol and agar. To remove the false positives on those areas, most of the teams created the mask for the kidney using different techniques such as Canny filters from OpenCV package and intensity thresholding. Since connectivity is one of the important factors for vascular trees and models failed in preserving these contextual long dependencies, connected component 3D (cc3d)^72^ was popularly used to improve the connectivity of the predictions as a post-processing method.

### Reporting summary

Further information on research design is available in the Nature Portfolio Reporting Summary linked to this article.

## Supplementary information

Supplementary Information

Reporting summary

Transparent Peer Review file

## Source data

Source data

## Data Availability

All training datasets are available on the competition website (https://www.kaggle.com/competitions/blood-vessel-segmentation/data). Additionally, complete competition dataset (including training datasets, test datasets, with ground truth labels), whole kidney datasets for test sets, additional VOI dataset, team predictions, and source data files for manuscript figures have been compiled into a single publicly available repository on Zenodo (10.5281/zenodo.19685381). The raw imaging datasets are also available, and can be visualized, on the Human Organ Atlas data portal at https://human-organ-atlas.esrf.eu. The DOIs to individual datasets are available in Supplementary Table 1. Source data for graphs in Fig. 2, Fig. 6, Supplementary Fig. 5, and Supplementary Fig. 6 are also provided as a Source Data File. Source data are provided with this paper.

## Code availability

All code for analysis and the winning solutions is publicly available on GitHub (https://github.com/cns-iu/hra-sennet-hoa-kaggle-2024). The code for morphological analysis is available on GitHub (https://github.com/HiPCTProject/Kaggle_skeleton_analyses). A snapshot of these repositories, including trained model weights for the top-5 team solutions, are also deposited into a single publicly available repository on Zenodo (10.5281/zenodo.19685078).
