# Supplementary material for: Vasculature segmentation in 3D hierarchical phase-contrast tomography images of human kidneys
Source: Nat Commun. 2026 Jul 3;17:8273. doi: 10.1038/s41467-026-74050-8 (PMC13470036; doi:10.1038/s41467-026-74050-8)
Supplement: Supplementary file 1 [file 41467_2026_74050_MOESM1_ESM.pdf]

Supplementary Information for Vasculature segmentation in 3D hierarchical phase-contrast tomography images of human kidneys

Yashvardhan Jain[1*+], Claire L. Walsh[2*+], Ekin Yagis[2,3], Shahab Aslani[2,4], Sonal Nandanwar[2], Yang Zhou[2], Juhyung Ha[1], Katherine S. Gustilo[1], Joseph Brunet[2,5], Shahrokh Rahmani[2,6], Paul Tafforeau[5], Alexandre Bellier[7], Griffin M. Weber[8], Peter D. Lee[2], Katy Börner[1*]

[1] Department of Intelligent Systems Engineering, Luddy School of Informatics, Computing, and Engineering, Indiana University, Bloomington, IN 47408, USA

[2] Department of Mechanical Engineering, University College London, London, UK

[3] Department of Surgery & Cancer - Faculty of Medicine Imperial College London, London UK

[4] UCL Centre for Medical Image Computing London UK

[5] European Synchrotron Radiation Facility, Grenoble, France

[6] National Heart and Lung Institute, Faculty of Medicine, Imperial College London, London, UK

[7] Univ. Grenoble Alpes, Department of Anatomy (LADAF), Grenoble, France

[8] Department of Biomedical Informatics, Harvard Medical School, Boston, MA, United States

[+]These authors contributed equally

*Corresponding authors

Yashvardhan Jain (yashjain@iu.edu)

Claire L. Walsh (c.walsh.11@ucl.ac.uk)

Katy Börner (katy@iu.edu)

Supplementary Figures

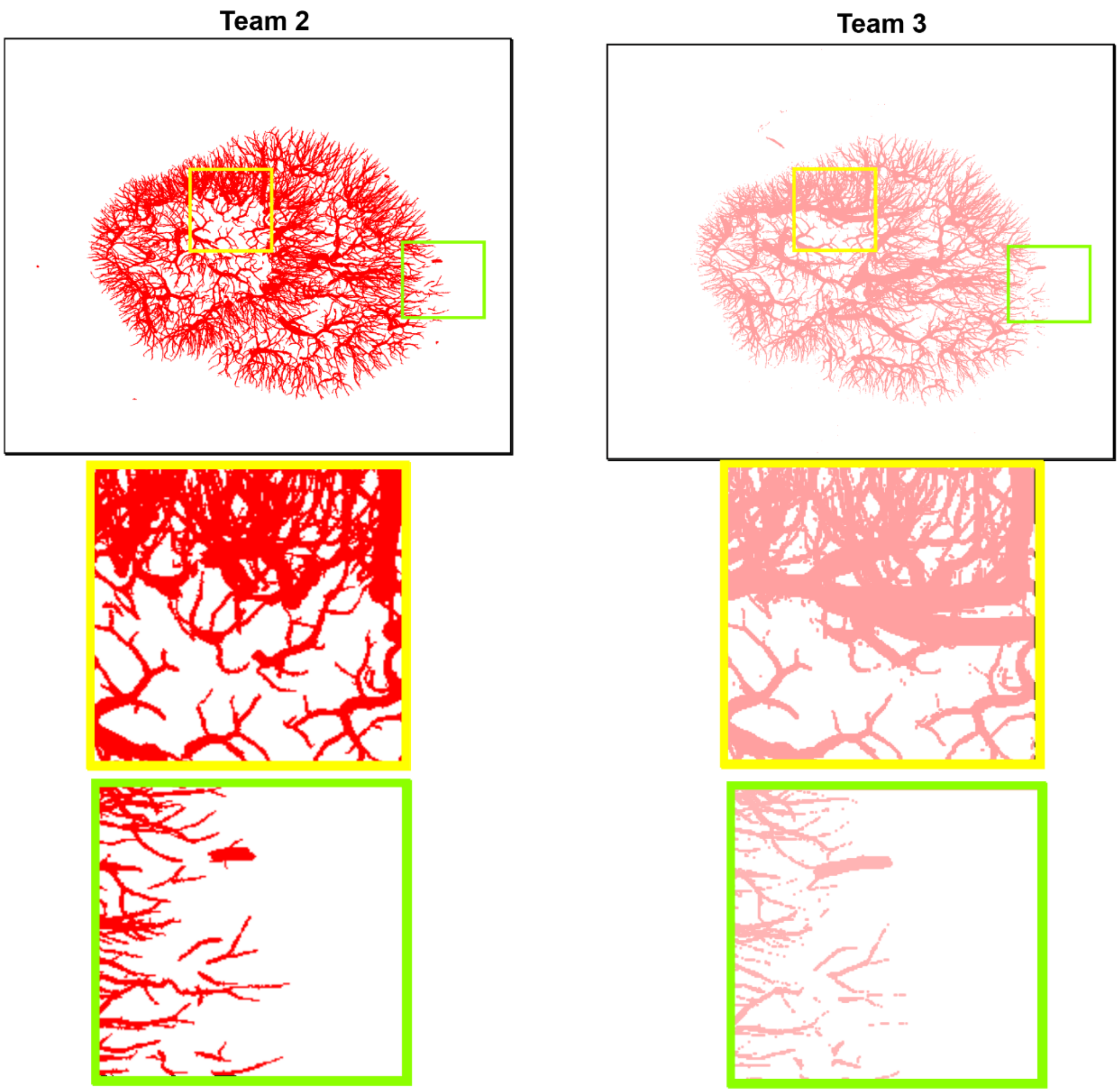

Supplementary Figure 1. Maximum Intensity Projections for Qualitative Analysis. Maximum intensity projections (MIP) for Teams 2 and 3, with two insets per team in the yellow and green squares, respectively.

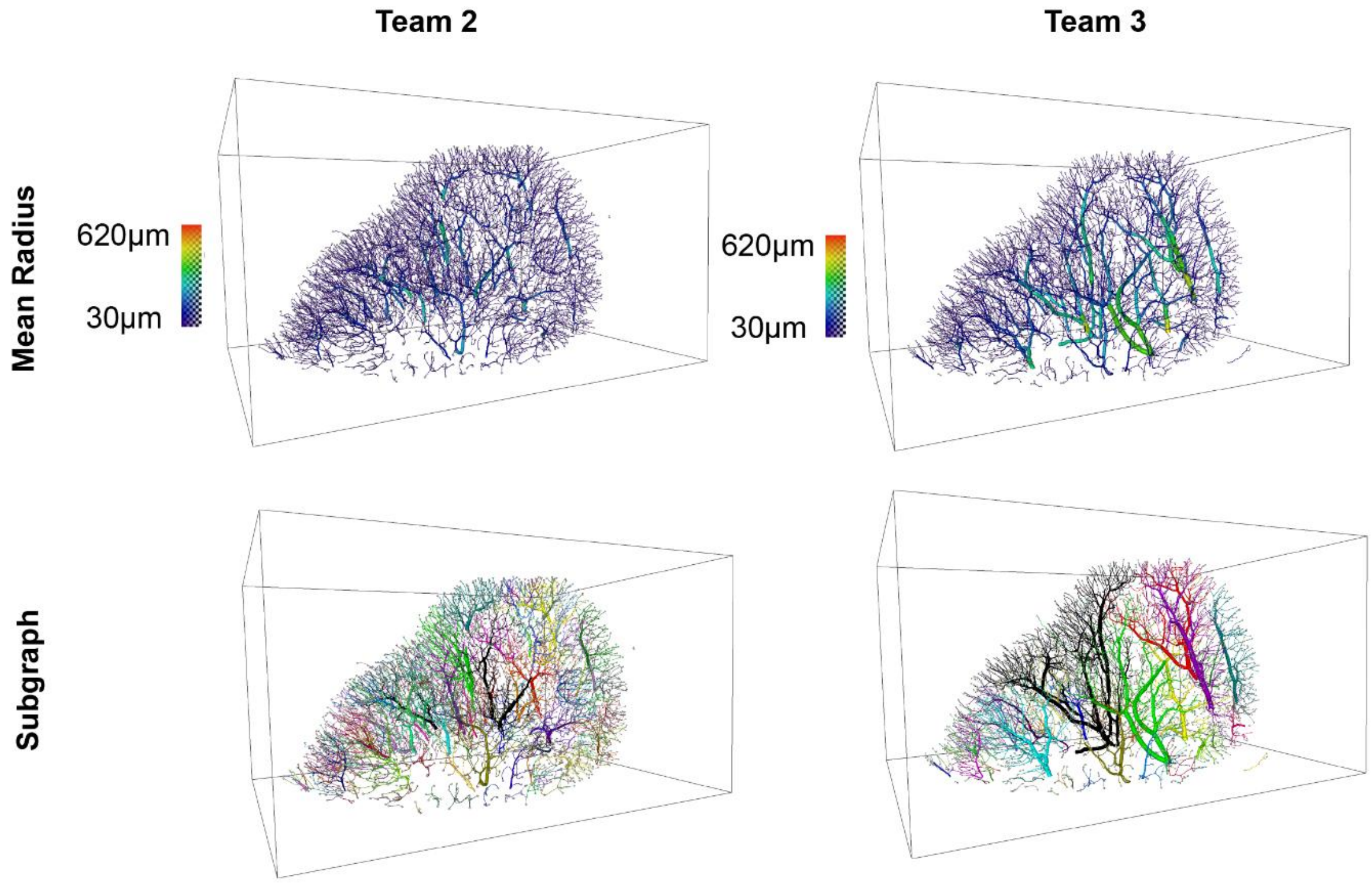

Supplementary Figure 2. Skeleton Visualization for Morphological Analysis. The figure shows the skeleton forms of the vessel network for Teams 2 and 3. In each case the vessel network size is proportional to the mean radius in the first row; the color shows the mean radius for each case. The second row is colored with each unconnected subgraph in a different color.

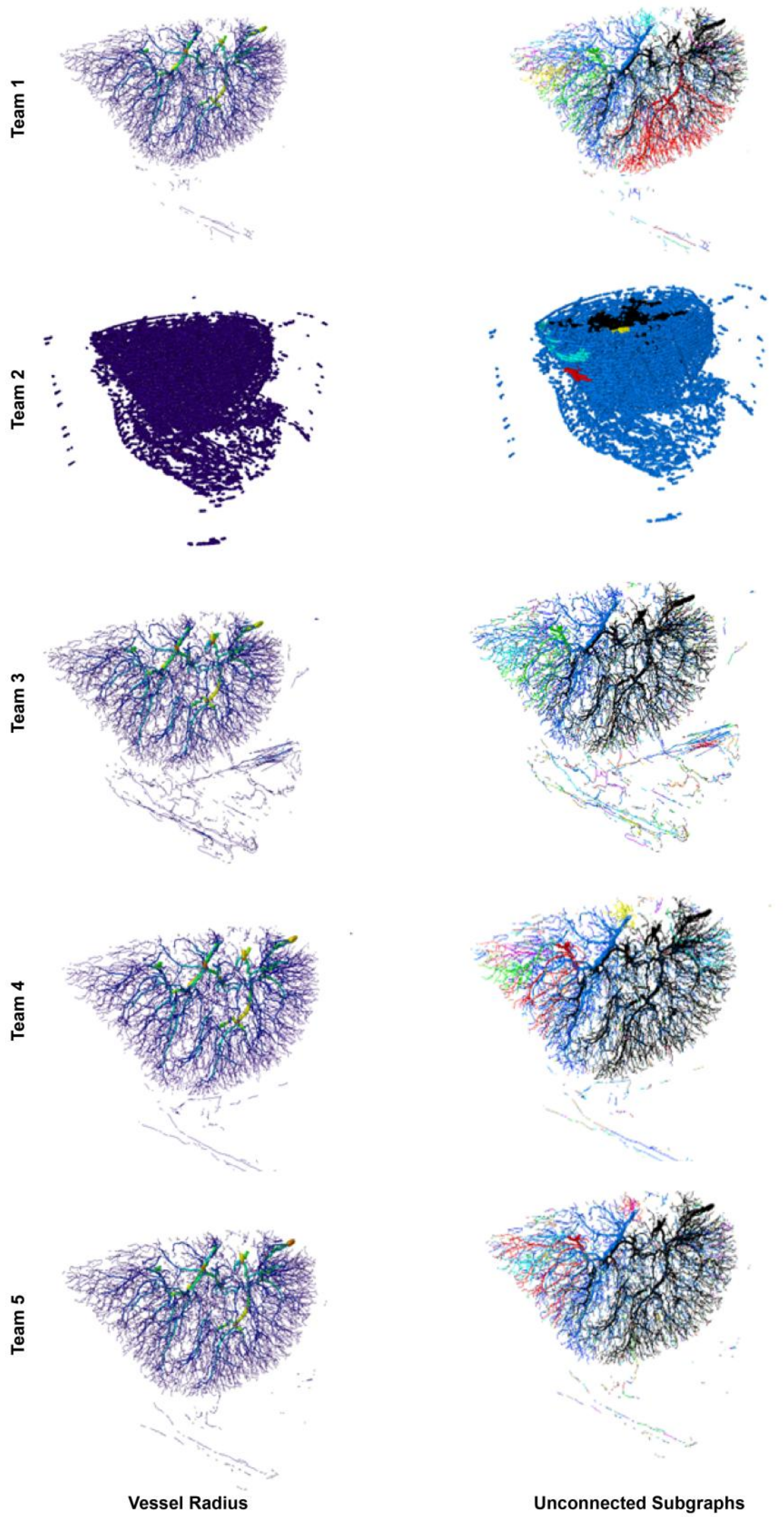
Team 1
Team 2
Team 3
Team 4
Team 5
Vessel Radius
Unconnected Subgraphs

Supplementary Figure 3. Skeleton Visualization for Morphological Analysis for Public Test Dataset. Showing the skeleton forms of the vessel network for all teams for the public test data. In each case the vessel network size is proportional to the mean radius in the first column; the color shows the mean radius for each case. The second column is colored with each unconnected subgraph in a different color.

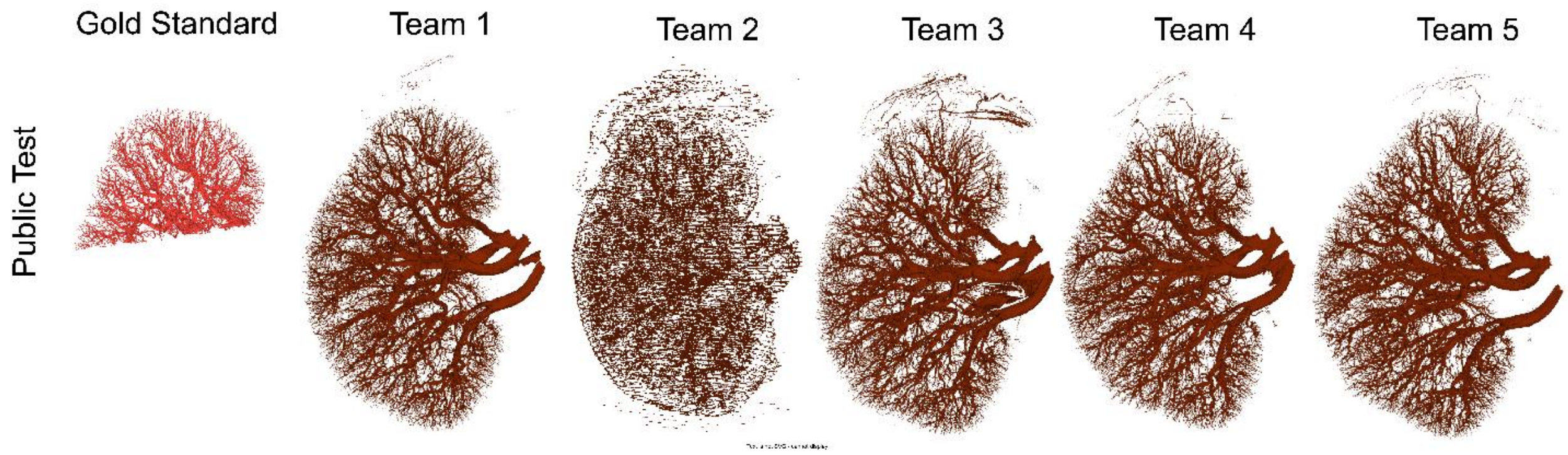

Supplementary Figure 4. Qualitative Analysis of the Vascular Trees for Public Test Dataset. Visualization of the 3D output for inference of each team on the whole intact kidney datasets for the public test data (Kidney 5). Gold standard (red) shows the part of the whole kidney that was fully labeled and was part of the original competition dataset.

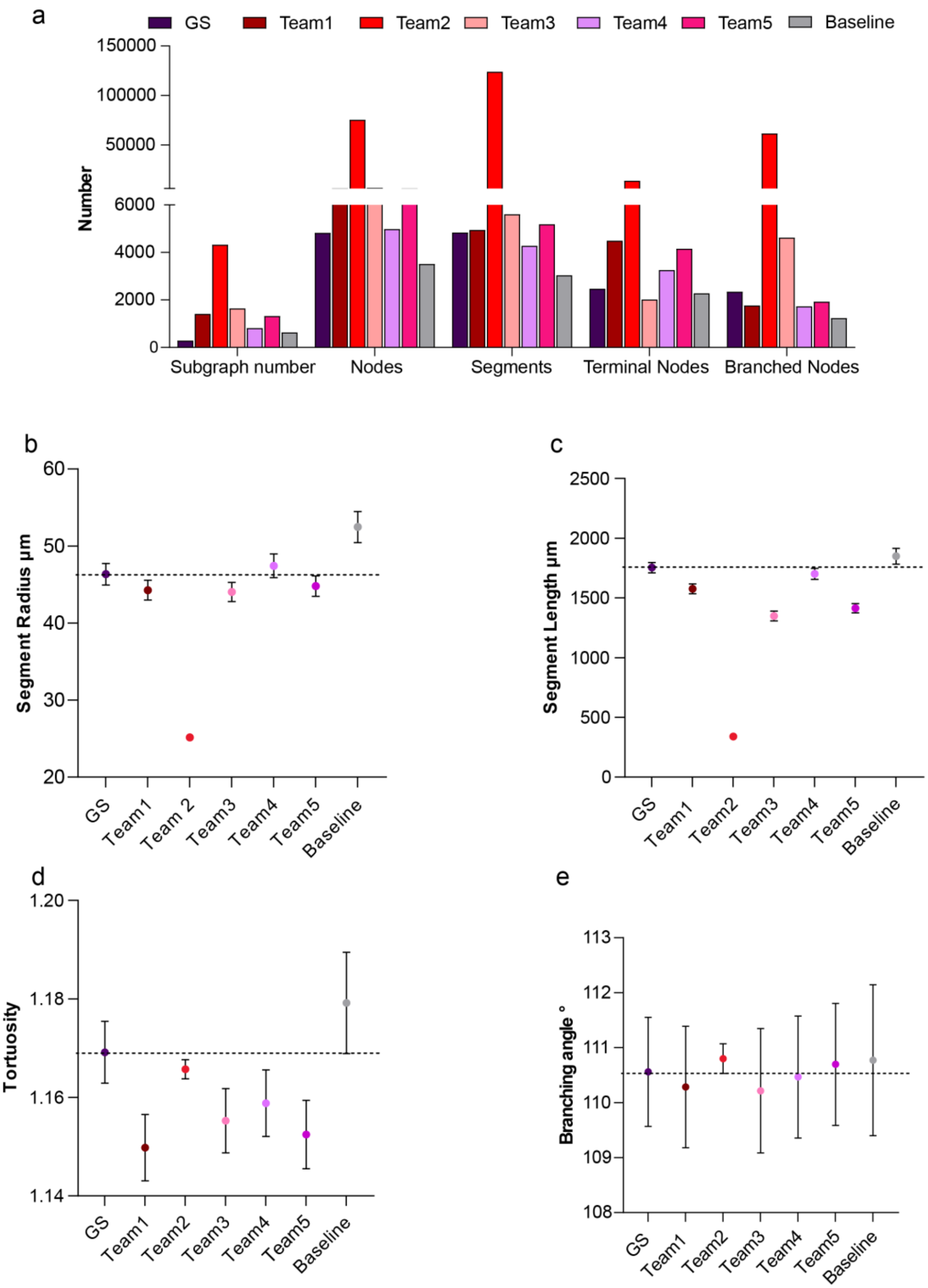
a
GS
Team1
Team2
Team3
Team4
Team5
Baseline
Number
150000
100000
50000
6000
4000
2000
0
Subgraph number
Nodes
Segments
Terminal Nodes
Branched Nodes
b
Segment Radius µm
60
50
40
30
20
GS
Team1
Team 2
Team3
Team4
Team5
Baseline
c
Segment Length µm
2500
2000
1500
1000
500
0
GS
Team1
Team2
Team3
Team4
Team5
Baseline
d
Tortuosity
1.20
1.18
1.16
1.14
GS
Team1
Team2
Team3
Team4
Team5
Baseline
e
Branching angle °
113
112
111
110
109
108
GS
Team1
Team2
Team3
Team4
Team5
Baseline

Supplementary Figure 5. Vascular Network Geometry for Morphological Analysis. a. Bar chart showing the number of subgraphs, nodes, segments, terminal nodes, and branched nodes for all team solutions as well as the baseline model predictions and gold standard (GS) labels for public test data. **b-e.** Plots showing the radius, length, tortuosity of segments, and the branching angle between segments; mean and 95% Confidence Interval (CI) are shown for all metrics. Source data are provided as a Source Data file (see **Data Availability**).

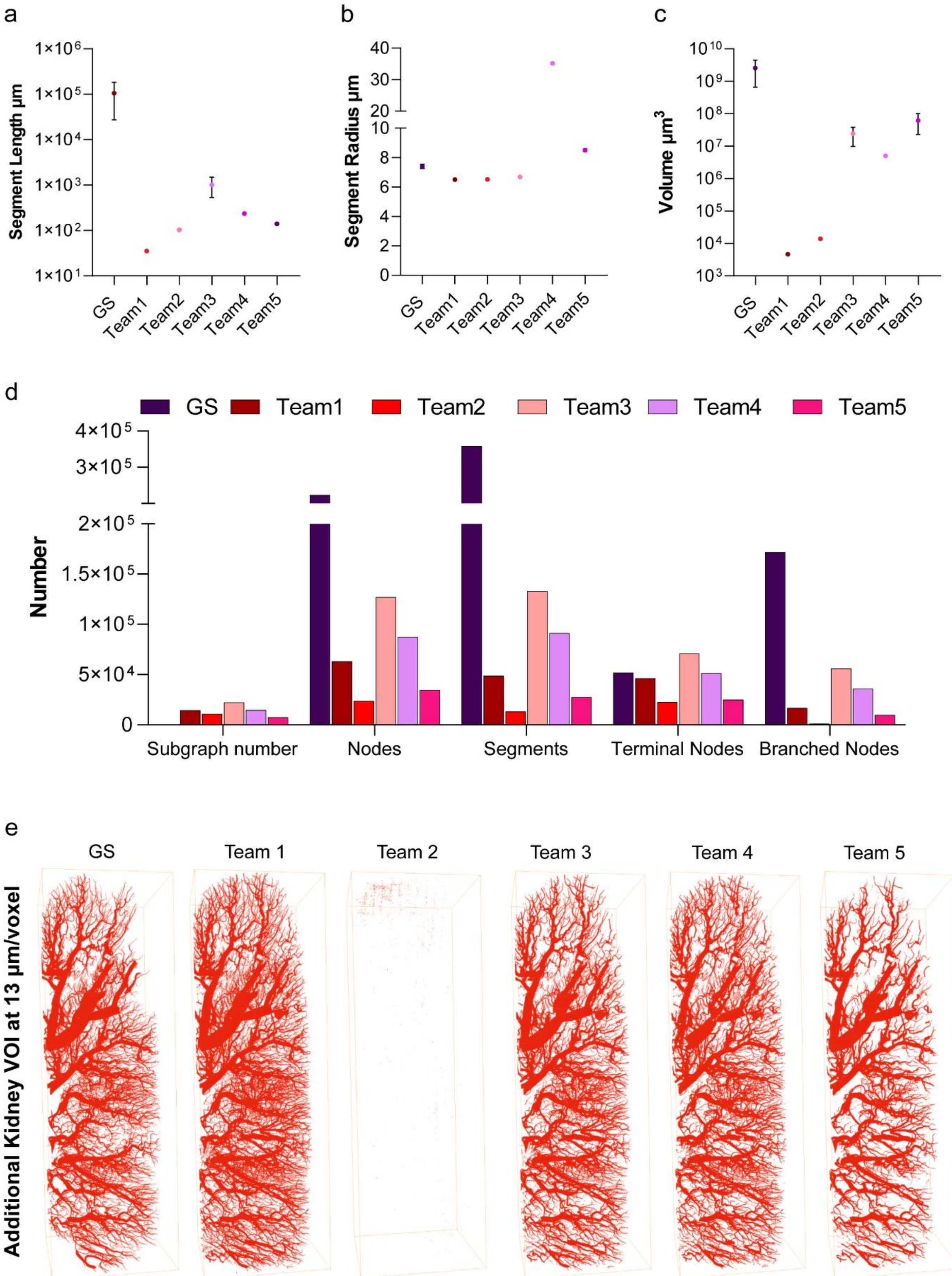

a
Segment Length µm
1×10^6
1×10^5
1×10^4
1×10^3
1×10^2
1×10^1
GS
Team1
Team2
Team3
Team4
Team5
b
Segment Radius µm
40
30
20
10
8
6
4
2
0
c
Volume µm^3
10^10
10^9
10^8
10^7
10^6
10^5
10^4
10^3
d
GS
Team1
Team2
Team3
Team4
Team5
Number
4×10^5
3×10^5
2×10^5
1.5×10^5
1×10^5
5×10^4
0
Subgraph number
Nodes
Segments
Terminal Nodes
Branched Nodes
e
GS
Team 1
Team 2
Team 3
Team 4
Team 5
Additional Kidney VOI at 13 µm/voxel

Supplementary Figure 6. Vascular Network Geometry and Vascular Tree for Additional VOI Dataset. a-c. Plots showing the segment length, segment radius, and the segment volume for additional dataset; mean and 95% Confidence Interval (CI) are shown for all metrics. **d.** Bar chart showing the number of subgraphs, nodes, segments, terminal nodes, and branched nodes for all team solutions and gold standard (GS) labels for additional dataset. **e.** Visualization of the Gold Standard (GS) labels and the 3D output for inference of each team on the additional dataset. Source data are provided as a Source Data file (see **Data Availability**).

Supplementary Tables

Supplementary Table 1. Complete Dataset Sources. Listing of DOI and link for each dataset in the competition data and the additional dataset.

Kaggle identifier	Donor identifier	Dataset name	DOI	Visualization link
**1**	LADAF-2021-17	LADAF-2021-17_kidney_right_complete-organ_25.0um_bm05	10.15151/ESRF-DC-1773966439	https://human-organ-atlas.esrf.eu/datasets/1773966586
**1**	LADAF-2021-17	LADAF-2021-17_kidney_right_VOI-03.1_2.6um_bm05	10.15151/ESRF-DC-2007535947	https://human-organ-atlas.esrf.fr/datasets/2008212318
**2**	S-20-28	S-20-28_kidney_complete-organ_25.0um_bm05	10.15151/ESRF-DC-2008212555	https://human-organ-atlas.esrf.fr/datasets/2008212321
**3**	LADAF-2020-27	LADAF-2020-27_kidney_left_complete-organ_25.08um_bm05	10.15151/ESRF-DC-572182553	https://human-organ-atlas.esrf.eu/datasets/572182201
**5**	LADAF-2021-17	LADAF-2021-17_kidney_left_complete-organ_25.14um_bm05	10.15151/ESRF-DC-1773965419	https://human-organ-atlas.esrf.eu/datasets/1773965003
**6**	LADAF-2022-13	LADAF-2022-13_kidney_2_complete-organ_15.77um_bm18	10.15151/ESRF-DC-2008212563	https://human-organ-atlas.esrf.fr/datasets/2008212345
**N/A (Additional Dataset, not used in the competition)**	LADAF-2021-17	LADAF-2021-17_kidney_right_VOI-03_6.5um	10.15151/ESRF-DC-2014789944	https://human-organ-atlas.esrf.fr/datasets/2016177220

Supplementary Table 2. Top-5 Teams Solution Overview. Scores for competition metric for top-5 teams and baseline model, including a brief summary.

Team	Summary of the model	Additional notes	Number of entries	Public score	Private score
1	Ensemble of two 2.5D convnext tiny U-net with 3 channels and custom loss	Data augmentation using random rotation	278	0.895	0.774
2	3D UNet with binary focal loss	Data augmentation using random rotation and post-processing to remove unconnected vessels	33	0.043	0.755
3	UNet with MaxViT-Large 512 backbone	Creating pseudo labels from dense labels (train on kidney 1 and kidney 3 (dense labels) to generate supplemental labels for kidney 3 sparse. Repeat label generation (step 2) for kidney 2 sparse	114	0.846	0.727
4	Ensemble of 2D and 3D UNet with d4 TTA using boundary DoU loss	Creating pseudo labels (an ensemble of 2d models trained with the same setup but without CutMix to create pseudo labels from additional data 50um_LADAF-2020-31)	251	0.884	0.712
5	Ensemble of 3 UNet models (effnet_v2_s, maxvit_base and dpn68) with custom loss (CE + Dice + Focal)	Creating pseudo labels (same models trained to create pseudo labels from additional data from human organ atlas -	108	0.855	0.691

		LADAF-2020-31 kidney and LADAF-2020-27 spleen)			
Baseline	NN-UNet Model	Default parameters	N/A	0.805	0.438

Supplementary Table 3. Quantitative Metrics for Top-50 Teams. All metric scores for top-50 teams, on both test sets. Team ID is based on ranking on the final private leaderboard. Lower is better for ASSD scores. Other metrics are bounded between 0 and 1, higher values are better.

Team ID	Dataset ID	NSD (t=0)	NSD (t=1)	clDice	ASSD	Dice
1	kidney_5	0.8951	0.9569	0.833	1.3132	0.9283
1	kidney_6	0.7741	0.9252	0.7859	0.7877	0.8289
2	kidney_5	0.0431	0.0855	0.0134	40.5078	0.0119
2	kidney_6	0.756	0.8479	0.7991	2.7445	0.6758
3	kidney_5	0.8327	0.8959	0.7493	10.8552	0.8971
3	kidney_6	0.7279	0.8844	0.7428	1.0919	0.8174
4	kidney_5	0.8849	0.9423	0.8457	1.8925	0.9296
4	kidney_6	0.7122	0.8623	0.7342	1.4183	0.8144
5	kidney_5	0.8855	0.9554	0.8279	1.1549	0.9279
5	kidney_6	0.6918	0.8612	0.7088	1.5988	0.8041
6	kidney_5	0.8862	0.9477	0.8411	0.7425	0.9315
6	kidney_6	0.6819	0.8055	0.6659	1.8331	0.792
7	kidney_5	0.8594	0.9156	0.7959	6.1061	0.9152
7	kidney_6	0.6768	0.8492	0.7139	1.4423	0.7802
8	kidney_5	0.879	0.9431	0.8088	1.4752	0.9275
8	kidney_6	0.6753	0.8585	0.7015	1.4384	0.778
9	kidney_5	0.8272	0.8998	0.7227	4.2324	0.9088
9	kidney_6	0.6651	0.8843	0.7343	1.1351	0.7758
10	kidney_5	0.8103	0.8795	0.7159	10.2946	0.8912
10	kidney_6	0.6572	0.8496	0.699	1.4754	0.7781
11	kidney_5	0.6132	0.7349	0.6529	17.6852	0.649
11	kidney_6	0.6559	0.7625	0.6824	3.2546	0.6951
12	kidney_5	0.6362	0.8144	0.5687	7.2457	0.801
12	kidney_6	0.6488	0.8012	0.6436	3.2204	0.7075
13	kidney_5	0.8581	0.9165	0.7899	3.1011	0.9138

13	kidney_6	0.6479	0.8382	0.6593	1.3991	0.7674
14	kidney_5	0	0		inf	0
14	kidney_6	0.6462	0.8177	0.6888	2.1497	0.7571
15	kidney_5	0.8228	0.9023	0.7518	4.6402	0.9011
15	kidney_6	0.6359	0.8184	0.6718	1.8454	0.7831
16	kidney_5	0.8887	0.9503	0.8065	1.8009	0.9285
16	kidney_6	0.6347	0.8635	0.6904	1.2871	0.7635
17	kidney_5	0.8137	0.8884	0.6991	2.1368	0.9077
17	kidney_6	0.6345	0.823	0.6739	1.8226	0.7782
18	kidney_5	0.8368	0.8961	0.7846	7.3325	0.8789
18	kidney_6	0.6245	0.8353	0.7032	5.0582	0.736
19	kidney_5	0.8133	0.8869	0.6914	1.3084	0.9074
19	kidney_6	0.6214	0.7885	0.6391	4.6257	0.724
20	kidney_5	0.8568	0.9287	0.7776	2.5528	0.9155
20	kidney_6	0.6167	0.8465	0.7333	1.4698	0.6847
21	kidney_5	0.8199	0.891	0.7248	7.8449	0.896
21	kidney_6	0.6141	0.8227	0.6838	2.1755	0.7271
22	kidney_5	0.8722	0.943	0.802	1.5679	0.921
22	kidney_6	0.6117	0.8155	0.6539	2.3056	0.7248
23	kidney_5	0.8507	0.9086	0.7707	1.6365	0.9179
23	kidney_6	0.6103	0.7729	0.654	3.0569	0.7025
24	kidney_5	0.8735	0.9414	0.831	2.6179	0.9188
24	kidney_6	0.6088	0.8202	0.6746	4.1682	0.7267
25	kidney_5	0	0		inf	0
25	kidney_6	0.608	0.8119	0.6585	1.9342	0.7667
26	kidney_5	0.8676	0.9379	0.7936	1.3814	0.9224
26	kidney_6	0.606	0.7712	0.6188	3.2132	0.7469
27	kidney_5	0.8652	0.924	0.8019	5.6499	0.9108
27	kidney_6	0.6035	0.7922	0.6504	2.6637	0.738

28	kidney_5	0.8496	0.9096	0.77	5.6527	0.9124
28	kidney_6	0.602	0.8053	0.6509	3.3284	0.7603
29	kidney_5	0.8594	0.9187	0.7875	1.2754	0.9178
29	kidney_6	0.5992	0.7264	0.594	3.1979	0.6882
30	kidney_5	0.8703	0.9338	0.805	2.6242	0.9242
30	kidney_6	0.5988	0.7907	0.6391	2.0989	0.7483
31	kidney_5	0.8301	0.9013	0.7501	2.9544	0.9069
31	kidney_6	0.5982	0.7992	0.6869	3.4409	0.7232
32	kidney_5	0.8561	0.9326	0.7795	1.5592	0.9157
32	kidney_6	0.5968	0.796	0.6446	2.1674	0.7349
33	kidney_5	0.8432	0.9163	0.7432	3.3218	0.9129
33	kidney_6	0.5959	0.7973	0.6252	6.5184	0.745
34	kidney_5	0.8754	0.9397	0.8121	1.9118	0.9235
34	kidney_6	0.5944	0.8322	0.6843	1.6091	0.7481
35	kidney_5	0.8503	0.9269	0.764	2.2443	0.9123
35	kidney_6	0.5943	0.7931	0.6531	3.0259	0.6511
36	kidney_5	0.8952	0.956	0.8509	0.7754	0.9322
36	kidney_6	0.5936	0.7696	0.609	2.7045	0.7683
37	kidney_5	0.6082	0.7485	0.5749	24.0377	0.7656
37	kidney_6	0.5905	0.7227	0.5769	6.9668	0.7079
38	kidney_5	0.6804	0.7684	0.6423	19.6523	0.7369
38	kidney_6	0.5905	0.7317	0.6036	5.5285	0.6497
39	kidney_5	0	0		inf	0
39	kidney_6	0.5896	0.8272	0.6714	1.733	0.7546
40	kidney_5	0.7982	0.8653	0.7004	11.4582	0.8858
40	kidney_6	0.5875	0.8007	0.639	2.9754	0.7554
41	kidney_5	0.8348	0.9147	0.7845	4.2633	0.9016
41	kidney_6	0.587	0.78	0.6293	4.6619	0.7572
42	kidney_5	0.8811	0.9477	0.815	0.8389	0.9261

42	kidney_6	0.5862	0.7822	0.6411	5.1143	0.6628
43	kidney_5	0.8711	0.9382	0.7985	1.7559	0.9229
43	kidney_6	0.5848	0.8085	0.6718	2.0455	0.6903
44	kidney_5	0.8761	0.9428	0.8073	1.9085	0.9241
44	kidney_6	0.5848	0.8102	0.6644	4.4716	0.6803
45	kidney_5	0.8941	0.9541	0.8403	0.7352	0.9297
45	kidney_6	0.584	0.827	0.6803	3.0314	0.6334
46	kidney_5	0.8065	0.8823	0.6973	2.8374	0.9005
46	kidney_6	0.5829	0.7626	0.6044	2.5261	0.7397
47	kidney_5	0.8283	0.9073	0.7383	3.4346	0.905
47	kidney_6	0.5824	0.7888	0.6594	2.2022	0.7001
48	kidney_5	0.8741	0.942	0.8172	0.8616	0.9241
48	kidney_6	0.5809	0.7819	0.6335	7.6572	0.7435
49	kidney_5	0.7685	0.8545	0.715	12.7754	0.8565
49	kidney_6	0.5788	0.738	0.6031	3.248	0.7292
50	kidney_5	0.8364	0.9029	0.7445	3.5469	0.9101
50	kidney_6	0.5782	0.799	0.6574	2.2052	0.7254

Supplementary Table 4. Quantitative Metrics for Top-5 Teams on Additional Dataset. All metric scores for top-5 teams, on additional dataset. Team ID is based on ranking on the final private leaderboard. Metrics are bounded between 0 and 1, higher values are better.

**Team ID**	**NSD (t=0)**	**NSD (t=1)**	**clDice**	**Dice**
1	0.7347	0.8430	0.7385	0.8983
2	0.0021	0.0033	0.0020	0.0003
3	0.7236	0.8297	0.7225	0.9006
4	0.6551	0.7681	0.6891	0.8493
5	0.4062	0.6287	0.5155	0.8570
