# Supplementary material for: Vasculature segmentation in 3D hierarchical phase-contrast tomography images of human kidneys
Source: Nat Commun. 2026 Jul 3;17:8273. doi: 10.1038/s41467-026-74050-8 (PMC13470036; doi:10.1038/s41467-026-74050-8)
Supplement: Supplementary file 2 [file 41467_2026_74050_MOESM2_ESM.pdf]

Corresponding author(s): Katy Börner
Claire L. Walsh
Yashvardhan Jain

Last updated by author(s): Apr 25, 2026

Reporting Summary

Nature Portfolio wishes to improve the reproducibility of the work that we publish. This form provides structure for consistency and transparency in reporting. For further information on Nature Portfolio policies, see our Editorial Policies and the Editorial Policy Checklist.

Statistics

For all statistical analyses, confirm that the following items are present in the figure legend, table legend, main text, or Methods section.

n/a	Confirmed	
☐	☒	The exact sample size (*n*) for each experimental group/condition, given as a discrete number and unit of measurement
☒	☐	A statement on whether measurements were taken from distinct samples or whether the same sample was measured repeatedly
☐	☒	The statistical test(s) used AND whether they are one- or two-sided<br>*Only common tests should be described solely by name; describe more complex techniques in the Methods section.*
☒	☐	A description of all covariates tested
☒	☐	A description of any assumptions or corrections, such as tests of normality and adjustment for multiple comparisons
☐	☒	A full description of the statistical parameters including central tendency (e.g. means) or other basic estimates (e.g. regression coefficient) AND variation (e.g. standard deviation) or associated estimates of uncertainty (e.g. confidence intervals)
☐	☒	For null hypothesis testing, the test statistic (e.g. *F*, *t*, *r*) with confidence intervals, effect sizes, degrees of freedom and *P* value noted<br>*Give P values as exact values whenever suitable.*
☒	☐	For Bayesian analysis, information on the choice of priors and Markov chain Monte Carlo settings
☒	☐	For hierarchical and complex designs, identification of the appropriate level for tests and full reporting of outcomes
☒	☐	Estimates of effect sizes (e.g. Cohen's *d*, Pearson's *r*), indicating how they were calculated

Our web collection on statistics for biologists contains articles on many of the points above.

Software and code

Policy information about availability of computer code

Data collection	All information about the data collection code is provided in the Code Availability statement and Methods. Gold standard label annotation is done using Amira-Avizo v2021.1.
Data analysis	All information about the analysis code is provided in the Code Availability statement and Methods. Kendall's Tau is computed using Python Scipy library. ASSD was computed using MONAI v1.3.0 library. Gold standard label annotation and morphological code analysis are done using Amira-Avizo v2021.1. All code for analysis and the winning solutions is publicly available on GitHub (https://github.com/cns-iu/hra-sennet-hoa-kaggle-2024). The code for morphological analysis is available on GitHub (https://github.com/HiPCTProject/Kaggle_skeleton_analyses). All code is also made available via zenodo (https://doi.org/10.5281/zenodo.19685078).

For manuscripts utilizing custom algorithms or software that are central to the research but not yet described in published literature, software must be made available to editors and reviewers. We strongly encourage code deposition in a community repository (e.g. GitHub). See the Nature Portfolio guidelines for submitting code & software for further information.

Data

Policy information about availability of data

All manuscripts must include a data availability statement. This statement should provide the following information, where applicable:

- Accession codes, unique identifiers, or web links for publicly available datasets
- A description of any restrictions on data availability
- For clinical datasets or third party data, please ensure that the statement adheres to our policy

All training datasets are available on the competition website (https://www.kaggle.com/competitions/blood-vessel-segmentation/data). Additionally, complete competition dataset (including training datasets, test datasets, with ground truth labels), whole kidney datasets for test sets, additional VOI dataset, team predictions, and source data files for manuscript figures have been compiled into a single publicly available repository on Zenodo (https://doi.org/10.5281/zenodo.19685381). The raw imaging datasets are also available, and can be visualized, on the Human Organ Atlas data portal at https://human-organ-atlas.esrf.eu. The DOIs to individual datasets are available in Supplementary Table 1.

Research involving human participants, their data, or biological material

Policy information about studies with human participants or human data. See also policy information about sex, gender (identity/presentation), and sexual orientation and race, ethnicity and racism.

Reporting on sex and gender	The work presents machine learning models for image segmentation on previously published imaging datasets. Due to the nature of the work, sex and/or gender are not relevant. All datasets are publicly available on Human Organ Atlas Hub. See Supplementary Table 1 for DOIs for each imaging dataset.
Reporting on race, ethnicity, or other socially relevant groupings	N/A
Population characteristics	N/A
Recruitment	All datasets used are previously published and publicly available on Human Organ Atlas Hub (https://human-organ-atlas.esrf.fr/). See Supplementary Table 1 for DOIs for each imaging dataset.
Ethics oversight	All datasets used are previously published and publicly available on Human Organ Atlas Hub (https://human-organ-atlas.esrf.fr/). See Supplementary Table 1 for DOIs for each imaging dataset. Names of boards/committees that approved the protocol:<br>1. Laboratoire d’Anatomie des Alpes Françaises<br>2. Hannover Institute of Pathology at Medizinische Hochschule, Hannover (Ethics vote no. 9621 BO K 2021).<br>3. French Health Ministry.

Note that full information on the approval of the study protocol must also be provided in the manuscript.

Field-specific reporting

Please select the one below that is the best fit for your research. If you are not sure, read the appropriate sections before making your selection.

☒ Life sciences ☐ Behavioural & social sciences ☐ Ecological, evolutionary & environmental sciences

For a reference copy of the document with all sections, see nature.com/documents/nr-reporting-summary-flat.pdf

Life sciences study design

All studies must disclose on these points even when the disclosure is negative.

Sample size	Sample size was determined by beamtime availability, and hours of expert annotation time. Data collection is detailed in the Methods section of the manuscript.
Data exclusions	No data were excluded. Data collection is detailed in the Methods section of the manuscript.
Replication	All winning algorithms were validated and compared to scores on competition leaderboard. This study reports on competition results and the total number of competition entries which would be equivalent to experimental repeats was 32,391
Randomization	4 samples were provided as training data and 2 as test data. An additional dataset was used in supplementary analysis.
Blinding	Blinding was not performed on test or training data for expert annotators. As the study was based on analysis of an open competition, this was not a necessary part of the study.

Reporting for specific materials, systems and methods

We require information from authors about some types of materials, experimental systems and methods used in many studies. Here, indicate whether each material, system or method listed is relevant to your study. If you are not sure if a list item applies to your research, read the appropriate section before selecting a response.

Materials & experimental systems

n/a	Involved in the study
☒	☐ Antibodies
☒	☐ Eukaryotic cell lines
☒	☐ Palaeontology and archaeology
☒	☐ Animals and other organisms
☒	☐ Clinical data
☒	☐ Dual use research of concern
☒	☐ Plants

Methods

n/a	Involved in the study
☒	☐ ChIP-seq
☒	☐ Flow cytometry
☒	☐ MRI-based neuroimaging

Plants

Seed stocks	N/A
Novel plant genotypes	N/A
Authentication	N/A
