# Supplementary material for: Vasculature segmentation in 3D hierarchical phase-contrast tomography images of human kidneys
Source: Nat Commun. 2026 Jul 3;17:8273. doi: 10.1038/s41467-026-74050-8 (PMC13470036; doi:10.1038/s41467-026-74050-8)
Supplement: Supplementary file 3 [file 41467_2026_74050_MOESM3_ESM.pdf]

nature portfolio

Peer Review File

Vasculature segmentation in 3D hierarchical phase-contrast tomography images of human kidneys

Corresponding Author: Mr Yashvardhan Jain

This file contains all reviewer reports in order by version, followed by all author rebuttals in order by version.

Version 0:

Reviewer comments:

Reviewer #2

(Remarks to the Author)
The article introduces a vascular segmentation competition in the field of medical image analysis and presents various methods to optimise segmentation accuracy. These include different network structures and data augmentation techniques to improve the effectiveness of training with small samples. The article also provides a reference benchmark for future segmentation tasks. However,the article presents a number of critical issues that require many improvements.The article is more akin to a report than an academic paper with depth and innovation. While it presents the design and outcomes of a global competition for vascular segmentation on 3D HiP-CT data, it lacks sufficient in-depth analysis and innovation. The main issues are as follows:

1. It is essential that the article provides more detailed explanations of the solutions adopted by each team, with particular emphasis on the specifics of parameter selection and boundary loss weight. A more comprehensive analysis of the problem-solving methodologies and critical perspectives is required.
2. The objective of this work is to enhance the generalisation capabilities of the proposed methodology. A more detailed examination of the obstacles encountered by Teams 2 and 3 in terms of model adaptability to unknown data is required. Further investigation and analysis of the issues surrounding generalization would be advantageous.
3. A discussion of the clinical application value is warranted. A further limitation of the study is the lack of in-depth discussion regarding the potential clinical application value of the proposed segmentation model in a clinical setting. It is recommended that further exploration be conducted into its potential practical application in the fields of vascular pathology research and treatment planning, with the aim of enhancing the research's utility and application orientation.
In conclusion, the article requires comprehensive strengthening in terms of analytical depth, innovation, and practical problem-solving aspects. It is recommended that the authors incorporate the above suggestions for improvement in order to enhance the research and make it more refined and academically valuable.

(Remarks on code availability)

Reviewer #3

(Remarks to the Author)
This manuscript reports on the results of a citizen science competition aimed at machine learning-based blood vessel segmentation in hierarchical phase-contrast tomography (HiP-CT) datasets of ex vivo human kidneys. The authors analysed the top-performing solutions – all based on variants of U-Net – finding that data augmentation and normalization, along with post-processing steps to ensure vessel connectivity, were important.

The authors list two purposes of this work: (I) establish “a benchmark for future studies in blood vessel segmentation within phase-contrast tomography imaging.” (Abstract), and (II) “develop open-source machine learning solutions capable of automatically segmenting large datasets to generate 3D models of the blood vasculature of the human kidney.” (Introduction). The authors’ ultimate goal “is to use the vascular segmentations to 1) model exosome transport, 2) extract vessel morphology metrics to create a VCCF” (Results).

Purpose I: It is unclear whether an “actual” benchmark can be obtained with this citizen science approach: 1) The

participants in the competition may lack the deep domain expertise necessary to fully exploit the nuances of HiP-CT imaging. 2) The competition format encourages participants to optimize solutions specifically for the given evaluation metric (i.e., NSD), which might not align perfectly with clinical or biological needs. The authors acknowledge this as the third point in their list of limitations. 3) Expert teams – domain experts (both for kidney anatomy and HiP-CT imaging) paired with experts in ML-based image segmentation methods – would likely have access to more and better resources (time, computing). Therefore, it is not clear to me how the presented benchmarks would compare to one set by domain experts. Maybe the authors can give examples from other areas where quantitative comparisons have been made.

Purpose II: It is unclear whether the developed ML solutions are of sufficient quality to meet the authors' ultimate goal. Most of the "interesting" transvascular transport in the kidney (and other organs) occurs at the capillary level, and there in the peritubular capillary network, which is not captured in the provided datasets. The peritubular capillary network poses formidable challenges to segmentation, since tubules of nephrons and blood vessels of comparable diameter run in very close proximity parallel to each other. Neural networks trained on blood vessel data alone will most likely not be able to differentiate between nephron tubules and capillaries. The authors could test the benchmark solutions on higher-resolution datasets that resolve tubules and capillaries.

That said, it is currently not possible to image an entire human kidney at the resolution needed to differentiate tubules and capillaries (in ex vivo kidneys, tubules and capillaries may touch, making a single epithelial-endothelial cell layer the critical structure to resolve). Whole-kidney datasets that resolve capillaries and tubules are available for the mouse in Scientific Data, 10, 510 (2023). These data were also acquired at ESRF (though at a different beamline and not using phase-contrast) and come with training data as well as a benchmark segmentation approach formulated by domain experts. For phase-contrast data acquired at ESRF, the authors could check Zdora et al., Optica, 7, 1221ff (2020), although I do not know what the data availability is. Alternatively, they could use the higher-resolution local tomography datasets that they already have and focus on the peritubular capillary network.

Given that we can expect imaging to advance and enable whole human organ acquisition at capillary resolution in the future, it would also be interesting to discuss how transfer learning could be applied, be it species to species in the same organ, or organ to organ in the human, modality to modality, or even combining these (e.g., selective plane illumination microscopy in the tissue-cleared mouse brain to HiP-CT in the human kidney).

Minor points:
Line 101: "Vessel segmentation is a challenging task for various reasons: it is an imbalanced class problem (where prediction targets are disproportionately lower in volume compared to non-target background)": I am not sure if I understand this correctly. If the authors are referring to the lower volume of labelled vs unlabelled structures, then this is more often than not a reflection of the limited resolution of imaging methods that do not allow capturing capillary blood vessels. If those are visible in the picture, then the labelled volume is quite large.

Line 125: Given that there was only one public test dataset, I assume that the "e.g." in this sentence should be an "i.e.".

Line 457: "segmenting both the very fine and the large the microvascular network": too many 'the's

(Remarks on code availability)

Version 1:

Reviewer comments:

Reviewer #2

(Remarks to the Author)
This manuscript, grounded in a large-scale Kaggle challenge, presents multiple teams' solutions for three-dimensional segmentation of renal vasculature in Hip-CT images. The work makes noteworthy contributions in dataset construction, comprehensive model summarization, and manuscript organization. However, as a submission to Nature Communications, it lacks a clear central thread of methodological innovation and a systematic, thorough evaluation of the models. The main points of concern are as follows:

The five summarized methods demonstrate good diversity, yet it is advisable to further clarify a principal line of methodological innovation. For instance, an in-depth investigation into the applicability and boundary conditions of a key approach—such as pseudo-labeling, self-training frameworks, or clDice topological loss—would substantially enhance the manuscript's impact.

In the qualitative evaluation of the models, exemplified by Figure 4a, it is recommended to provide more detailed comparisons highlighting differences in the performance of the five models, including aspects such as microvascular breaks, false connections, and under-segmentation, accompanied by error analyses to deepen readers' understanding of model limitations.

Throughout the model evaluation, it would be beneficial to supplement with in-depth analyses of methodological robustness,

including resistance to imaging artifacts and performance distribution across different renal regions (e.g., cortex, medulla, and hilum). For Figure 2c, a thorough analysis connecting the models' methodological characteristics to their generalization performance is suggested. Moreover, the 3D segmentation results would benefit from medical interpretability assessments, such as expert blinded reviews.

(Remarks on code availability)

Reviewer #4

(Remarks to the Author)
Given the unavailbility of one of the referee's I have been invited as a new reviewer to assess the revised manuscript. So I took the perspective of a clinician-scientist who is interested in this biology (and less an expert in bioinformatics). The whole approach for building deep learning models by organizing a multidisciplinary citizen science competition in itsself is very unconventional but highly innovative (and arguably the most innovative aspect of the paper). While the reviewers raise some concerns with respect to domain expertise the solutions generated look very convincing. From a clinical perspective the future application of such solutions could be very helpful in CKD and transplant organ risk stratification, so it definately is of relevance. Finally a strong asset is collection of expert curated datasets with gold standard segmentations for future studies; this is an aspect that is often overlooked and undervalued in ML approaches and this also counterbalances perhaps the lack of domain expertise of the contesters.

(Remarks on code availability)

Version 2:

Reviewer comments:

Reviewer #2

(Remarks to the Author)
I have no further comment.

(Remarks on code availability)

Open Access This Peer Review File is licensed under a Creative Commons Attribution 4.0 International License, which permits use, sharing, adaptation, distribution and reproduction in any medium or format, as long as you give appropriate credit to the original author(s) and the source, provide a link to the Creative Commons license, and indicate if changes were made.
In cases where reviewers are anonymous, credit should be given to 'Anonymous Referee' and the source.

The images or other third party material in this Peer Review File are included in the article's Creative Commons license, unless indicated otherwise in a credit line to the material. If material is not included in the article's Creative Commons license and your intended use is not permitted by statutory regulation or exceeds the permitted use, you will need to obtain permission directly from the copyright holder.
To view a copy of this license, visit https://creativecommons.org/licenses/by/4.0/

Author Response to Reviewer Comments

Reviewer #2 (Remarks to the Author):

The article introduces a vascular segmentation competition in the field of medical image analysis and presents various methods to optimise segmentation accuracy. These include different network structures and data augmentation techniques to improve the effectiveness of training with small samples. The article also provides a reference benchmark for future segmentation tasks. However, the article presents a number of critical issues that require many improvements.The article is more akin to a report than an academic paper with depth and innovation. While it presents the design and outcomes of a global competition for vascular segmentation on 3D HiP-CT data, it lacks sufficient in-depth analysis and innovation. The main issues are as follows:

1. It is essential that the article provides more detailed explanations of the solutions adopted by each team, with particular emphasis on the specifics of parameter selection and boundary loss weight. A more comprehensive analysis of the problem-solving methodologies and critical perspectives is required.

Author Response: We expanded the technical details of the winning solutions in the **Supplementary Information Notes 1-5**. We did not add this technical information to the main paper to make the text accessible to the wider multidisciplinary audience of *Nature Communications*. However, we have provided an extended critical analysis via comparing all 5 solutions to a new dataset, as detailed below.

2. The objective of this work is to enhance the generalisation capabilities of the proposed methodology. A more detailed examination of the obstacles encountered by Teams 2 and 3 in terms of model adaptability to unknown data is required. Further investigation and analysis of the issues surrounding generalization would be advantageous.

Author Response: We added an "additional dataset" (different resolution data from the same kidney as training data) and have tested all five solutions on the dataset. The results are reflected in the newly added **Supplementary Table 4** and **Supplementary Figure 6,** as well as the expanded **Results** (Quantitative and Morphological analysis) and **Discussion** sections. We also added further discussion of skeletonization challenges and the invariability in morphological metrics that can arise from inaccuracies in the skeletonization algorithms.

3. A discussion of the clinical application value is warranted. A further limitation of the study is the lack of in-depth discussion regarding the potential clinical application value of the proposed segmentation model in a clinical setting. It is recommended that further exploration be conducted into its potential practical application in the fields of vascular pathology research and treatment planning, with the aim of enhancing the research's utility and application orientation.

Author Response: While a discussion of the clinical application of segmentation models or HiP-CT can be interesting, it is beyond the scope of this work. Our focus here is on flow modeling tasks–such as blood flow, drug delivery, and exosome transport–and we have elaborated this point in the **Introduction** for better clarity.

In conclusion, the article requires comprehensive strengthening in terms of analytical depth, innovation, and practical problem-solving aspects. It is recommended that the authors incorporate the above suggestions for improvement in order to enhance the research and make it more refined and academically valuable.

Author Response: We have added a new dataset for further testing of the models and have updated the text to address the concerns mentioned above. We have revised the **Abstract** and **Introduction** to make it easier to understand that the paper presents:

- Different techniques for building deep learning models by organizing a citizen science competition for the defined segmentation task.
- Different relevant metrics to assess such algorithms, specifically for vascular segmentation in HiP-CT imaging and for flow modeling purposes.
- A collection of expert curated datasets with gold standard segmentations for future studies and competitions in blood vessel segmentation within HiP-CT imaging which is a new data modality with a rapidly increasing number of datasets being published on the Human Organ Atlas portal.

Reviewer #3 (Remarks to the Author):

This manuscript reports on the results of a citizen science competition aimed at machine learning-based blood vessel segmentation in hierarchical phase-contrast tomography (HiP-CT) datasets of ex vivo human kidneys. The authors analysed the top-performing solutions – all based on variants of U-Net – finding that data augmentation and normalization, along with post-processing steps to ensure vessel connectivity, were important.

The authors list two purposes of this work: (I) establish "a benchmark for future studies in blood vessel segmentation within phase-contrast tomography imaging." (Abstract), and

(II) “develop open-source machine learning solutions capable of automatically segmenting large datasets to generate 3D models of the blood vasculature of the human kidney.” (Introduction). The authors’ ultimate goal “is to use the vascular segmentations to 1) model exosome transport, 2) extract vessel morphology metrics to create a VCCF” (Results).

Purpose I: It is unclear whether an “actual” benchmark can be obtained with this citizen science approach: 1) The participants in the competition may lack the deep domain expertise necessary to fully exploit the nuances of HiP-CT imaging. 2) The competition format encourages participants to optimize solutions specifically for the given evaluation metric (i.e., NSD), which might not align perfectly with clinical or biological needs. The authors acknowledge this as the third point in their list of limitations. 3) Expert teams – domain experts (both for kidney anatomy and HiP-CT imaging) paired with experts in ML-based image segmentation methods – would likely have access to more and better resources (time, computing). Therefore, it is not clear to me how the presented benchmarks would compare to one set by domain experts. Maybe the authors can give examples from other areas where quantitative comparisons have been made.

Author Response: We have updated the text to reflect that this work does not aim to provide a “benchmark” in terms of datasets or machine learning models, but rather aims to present:

- Different techniques for building deep learning models by organizing a citizen science competition for the defined segmentation task.
- Different relevant metrics to assess such algorithms, specifically for vascular segmentation in HiP-CT imaging and for flow modeling purposes.
- A collection of expert curated datasets with gold standard segmentations for future studies and competitions in blood vessel segmentation within HiP-CT imaging which is a new data modality with a rapidly increasing number of datasets being published on the Human Organ Atlas portal.

While it is true that most participants may lack the deep domain expertise, most participants and teams at the top of the leaderboards are machine learning experts, either in industry or academia, with deep knowledge of machine learning and data science in various domains. Additionally, the nature of a competition organized on Kaggle provides a public forum (https://www.kaggle.com/competitions/blood-vessel-segmentation/discussion) to the community to constantly share their findings throughout the run of the competition which enables all participants to develop better solutions. That being said, we agree it might not be accurate to refer to these models as “benchmarks.” Instead, we have updated the paper text to reflect that the work presents a “template”

regarding development of segmentation models for HiP-CT imaging, rather than a "benchmark."

Purpose II: It is unclear whether the developed ML solutions are of sufficient quality to meet the authors' ultimate goal. Most of the "interesting" transvascular transport in the kidney (and other organs) occurs at the capillary level, and there in the peritubular capillary network, which is not captured in the provided datasets. The peritubular capillary network poses formidable challenges to segmentation, since tubules of nephrons and blood vessels of comparable diameter run in very close proximity parallel to each other. Neural networks trained on blood vessel data alone will most likely not be able to differentiate between nephron tubules and capillaries. The authors could test the benchmark solutions on higher-resolution datasets that resolve tubules and capillaries.

That said, it is currently not possible to image an entire human kidney at the resolution needed to differentiate tubules and capillaries (in ex vivo kidneys, tubules and capillaries may touch, making a single epithelial-endothelial cell layer the critical structure to resolve). Whole-kidney datasets that resolve capillaries and tubules are available for the mouse in Scientific Data, 10, 510 (2023). These data were also acquired at ESRF (though at a different beamline and not using phase-contrast) and come with training data as well as a benchmark segmentation approach formulated by domain experts. For phase-contrast data acquired at ESRF, the authors could check Zdora et al., Optica, 7, 1221ff (2020), although I do not know what the data availability is. Alternatively, they could use the higher-resolution local tomography datasets that they already have and focus on the peritubular capillary network.

Author Response: We have now added another "additional dataset" (different resolution data from the same kidney as training data) and have tested all five solutions on the dataset. The results are reflected in the newly added **Supplementary Table 4** and **Supplementary Figure 6,** as well as the expanded **Results** (Quantitative and Morphological analysis) and **Discussion** sections. We also added further discussion of skeletonization challenges and the invariability in morphological metrics that can arise from inaccuracies in the skeletonization algorithms. We agree that while it is currently not possible to image an entire human kidney at the resolution needed to differentiate tubules and capillaries, primarily being a limitation of the imaging technology than that of machine learning models, the approaches and insights gained through this competition are still relevant and useful for arterial segmentation in other kidney HiP-CT datasets and the future development of machine learning models as HiP-CT datasets increase, in resolution and quantity.

Given that we can expect imaging to advance and enable whole human organ acquisition at capillary resolution in the future, it would also be interesting to discuss how transfer learning could be applied, be it species to species in the same organ, or organ to organ in

the human, modality to modality, or even combining these (e.g., selective plane illumination microscopy in the tissue-cleared mouse brain to HiP-CT in the human kidney).

Author Response: The authors agree that transfer learning approaches would be a very interesting addition to this manuscript and will indeed play a crucial role as more higher resolution datasets become available, but at this time this is beyond the scope of the paper since this would require much more time and resource investment for generating more labeled gold standard data to properly validate the results. The authors added this as future work to the **Discussion** section.

Minor points:
Line 101: "Vessel segmentation is a challenging task for various reasons: it is an imbalanced class problem (where prediction targets are disproportionately lower in volume compared to non-target background)": I am not sure if I understand this correctly. If the authors are referring to the lower volume of labelled vs unlabelled structures, then this is more often than not a reflection of the limited resolution of imaging methods that do not allow capturing capillary blood vessels. If those are visible in the picture, then the labelled volume is quite large.

Author Response: Here, we are specifically referring to HiP-CT imaging datasets as used in the competition data, where due to the limited resolution, the prediction targets are disproportionately lower in volume compared to non-target background. We have edited the text to reflect this.

Line 125: Given that there was only one public test dataset, I assume that the "e.g." in this sentence should be an "i.e.".

Author Response: We have edited the text to correct this.

Line 457: "segmenting both the very fine and the large the microvascular network": too many 'the's

Author Response: We have edited the text to correct this.

Author Response to Reviewer Comments

Reviewer #2 (Remarks to the Author):

This manuscript, grounded in a large-scale Kaggle challenge, presents multiple teams' solutions for three-dimensional segmentation of renal vasculature in Hip-CT images. The work makes noteworthy contributions in dataset construction, comprehensive model summarization, and manuscript organization. However, as a submission to Nature Communications, it lacks a clear central thread of methodological innovation and a systematic, thorough evaluation of the models. The main points of concern are as follows:

The five summarized methods demonstrate good diversity, yet it is advisable to further clarify a principal line of methodological innovation. For instance, an in-depth investigation into the applicability and boundary conditions of a key approach—such as pseudo-labeling, self-training frameworks, or clDice topological loss—would substantially enhance the manuscript's impact.

Author Response: We have expanded the "Overview of winning solutions" section in **Results** to better reflect the methodological innovations and boundary conditions.

In the qualitative evaluation of the models, exemplified by Figure 4a, it is recommended to provide more detailed comparisons highlighting differences in the performance of the five models, including aspects such as microvascular breaks, false connections, and under-segmentation, accompanied by error analyses to deepen readers' understanding of model limitations.

Author Response: This section has been extended to link the microvascular breaks to model architecture and training paradigm, and an extra section has been added to focus on larger vessel misclassification error.

Throughout the model evaluation, it would be beneficial to supplement with in-depth analyses of methodological robustness, including resistance to imaging artifacts and performance distribution across different renal regions (e.g., cortex, medulla, and hilum).

Author Response: We have extended this section and provided a new figure (Figure 4) with qualitative discussion around model performance in the larger vessels of the kidney pelvis as compared to the microvascular characterization of the previous section and figure (Figure 3). The "Overview of winning solutions" also connects the architectural and training

methodologies to overcoming ring artefacts in computed tomography and to intensity heterogeneity in HiP-CT imaging.

For Figure 2c, a thorough analysis connecting the models' methodological characteristics to their generalization performance is suggested.

Author Response: We have expanded the "Quantitative analysis of winning solutions" section in **Results** to better reflect the methodological characteristics to figure 2c.

Moreover, the 3D segmentation results would benefit from medical interpretability assessments, such as expert blinded reviews.
Author Response: As HiP-CT provides ex vivo human kidney imaging at approximately an order of magnitude higher spatial resolution than current clinical imaging modalities, the most meaningful route to medical interpretability in this context is not limited to expert visual inspection alone, but through quantitative structural modeling. In our previous work, we demonstrated that skeletonization and flow-based analyses derived from segmented vascular networks enable physiologically relevant modeling of blood flow, drug delivery, and exosome transport (Y. Janvanmardi et al. 2025). Crucially, such downstream modeling requires segmentations that preserve global connectivity and accurately delineate large-caliber vessels that dominate hemodynamic resistance. We have revised the manuscript to explicitly connect the segmentation performance to these clinically relevant modeling requirements through the discussion and the updated result section. Additionally, while a formal blinded expert review was not performed, renal physiology and vascular specialists were an integral part of the competition organization and authorship team. This interdisciplinary oversight informed the competition setup and interpretation of the results.

Reviewer #4 (Remarks to the Author):

Given the unavailbility of one of the referee's I have been invited as a new reviewer to assess the revised manuscript. So I took the perspective of a clinician-scientist who is interested in this biology (and less an expert in bioinformatics). The whole approach for building deep learning models by organizing a multidisciplinary citizen science competition in itsself is very unconventional but highly innovative (and arguably the most innovative aspect of the paper). While the reviewers raise some concerns with respect to domain expertise the solutions generated look very convincing. From a clinical perspective the future application of such solutions could be very helpful in CKD and transplant organ risk stratification, so it definately is of relevance. Finally a strong asset is collection of expert curated datasets with gold standard segmentations for future studies; this is an aspect that is often overlooked and undervalued in ML approaches and this also counterbalances perhaps the lack of domain expertise of the contesters.

Author Response: We are glad to see the reviewer’s strong endorsement of this work.
